# GWAS meta-analysis reveals novel loci and genetic correlates for general cognitive function: a report from the COGENT consortium

**DOI:** 10.1038/mp.2016.244

**Published:** 2017-01-17

**Authors:** J W Trampush, M L Z Yang, J Yu, E Knowles, G Davies, D C Liewald, J M Starr, S Djurovic, I Melle, K Sundet, A Christoforou, I Reinvang, P DeRosse, A J Lundervold, V M Steen, T Espeseth, K Räikkönen, E Widen, A Palotie, J G Eriksson, I Giegling, B Konte, P Roussos, S Giakoumaki, K E Burdick, A Payton, W Ollier, M Horan, O Chiba-Falek, D K Attix, A C Need, E T Cirulli, A N Voineskos, N C Stefanis, D Avramopoulos, A Hatzimanolis, D E Arking, N Smyrnis, R M Bilder, N A Freimer, T D Cannon, E London, R A Poldrack, F W Sabb, E Congdon, E D Conley, M A Scult, D Dickinson, R E Straub, G Donohoe, D Morris, A Corvin, M Gill, A R Hariri, D R Weinberger, N Pendleton, P Bitsios, D Rujescu, J Lahti, S Le Hellard, M C Keller, O A Andreassen, I J Deary, D C Glahn, A K Malhotra, T Lencz

**Affiliations:** 1 Division of Psychiatry Research, Zucker Hillside Hospital, Glen Oaks, NY, USA; 2 Institute of Mental Health, Singapore, Singapore; 3 Center for Psychiatric Neuroscience, Feinstein Institute for Medical Research, Manhasset, NY, USA; 4 Department of Psychiatry, Yale University School of Medicine, New Haven, CT, USA; 5 Centre for Cognitive Ageing and Cognitive Epidemiology, University of Edinburgh, Edinburgh, UK; 6 Department of Psychology, University of Edinburgh, Edinburgh, UK; 7 Alzheimer Scotland Dementia Research Centre, University of Edinburgh, Edinburgh, UK; 8 Department of Medical Genetics, Oslo University Hospital, University of Bergen, Oslo, Norway; 9 NORMENT, K.G. Jebsen Centre for Psychosis Research, University of Bergen, Bergen, Norway; 10 Division of Mental Health and Addiction, Oslo University Hospital, Oslo, Norway; 11 Department of Psychology, University of Oslo, Oslo, Norway; 12 Dr Einar Martens Research Group for Biological Psychiatry, Center for Medical Genetics and Molecular Medicine, Haukeland University Hospital, Bergen, Norway; 13 Department of Biological and Medical Psychology, University of Bergen, Bergen, Norway; 14 Institute of Behavioural Sciences, University of Helsinki, Helsinki, Finland; 15 Institute for Molecular Medicine Finland (FIMM), University of Helsinki, Helsinki, Finland; 16 Wellcome Trust Sanger Institute, Wellcome Trust Genome Campus, Cambridge, UK; 17 Department of Medical Genetics, University of Helsinki and University Central Hospital, Helsinki, Finland; 18 National Institute for Health and Welfare, Helsinki, Finland; 19 Department of General Practice and Primary Health Care, University of Helsinki, Helsinki, Finland; 20 Helsinki University Central Hospital, Unit of General Practice, Helsinki, Finland; 21 Folkhälsan Research Centre, Helsinki, Finland; 22 Department of Psychiatry, Martin Luther University of Halle-Wittenberg, Halle, Germany; 23 Department of Psychiatry, Icahn School of Medicine at Mount Sinai, New York, NY, USA; 24 Department of Genetics and Genomic Science and Institute for Multiscale Biology, Icahn School of Medicine at Mount Sinai, New York, NY, USA; 25 Mental Illness Research, Education, and Clinical Center (VISN 3), James J. Peters VA Medical Center, Bronx, NY, USA; 26 Department of Psychology, University of Crete, Rethymno, Greece; 27 Manchester Centre for Audiology and Deafness, Manchester Academic Health Science Centre, The University of Manchester, Manchester, UK; 28 Division of Evolution and Genomic Sciences, School of Biological Sciences, The University of Manchester, Manchester, UK; 29 Centre for Integrated Genomic Medical Research, Institute of Population Health, University of Manchester, Manchester, UK; 30 Manchester Medical School, Institute of Brain, Behaviour, and Mental Health, University of Manchester, Manchester, UK; 31 Department of Neurology, Bryan Alzheimer's Disease Research Center, and Center for Genomic and Computational Biology, Duke University Medical Center, Durham, NC, USA; 32 Division of Medical Psychology, Department of Neurology, Psychiatry and Behavioral Sciences, Duke University Medical Center, Durham, NC, USA; 33 Division of Brain Sciences, Department of Medicine, Imperial College, London, UK; 34 Center for Applied Genomics and Precision Medicine, Duke University School of Medicine, Durham, NC, USA; 35 Campbell Family Mental Health Institute, Centre for Addiction and Mental Health, University of Toronto, Toronto, ON, Canada; 36 Department of Psychiatry, University of Athens School of Medicine, Eginition Hospital, Athens, Greece; 37 University Mental Health Research Institute, Athens, Greece; 38 Neurobiology Research Institute, Theodor Theohari Cozzika Foundation, Athens, Greece; 39 Department of Psychiatry, Johns Hopkins University School of Medicine, Baltimore, MD, USA; 40 Department of Psychiatry and McKusick-Nathans Institute of Genetic Medicine, Johns Hopkins University School of Medicine, Baltimore, MD, USA; 41 UCLA Semel Institute for Neuroscience and Human Behavior, Los Angeles, CA, USA; 42 Department of Psychology, Yale University, New Haven, CT, USA; 43 Department of Psychology, Stanford University, Palo Alto, CA, USA; 44 Robert and Beverly Lewis Center for Neuroimaging, University of Oregon, Eugene, OR, USA; 45 23andMe, Inc., Mountain View, CA, USA; 46 Department of Psychology & Neuroscience, Laboratory of NeuroGenetics, Duke University, Durham, NC, USA; 47 Clinical and Translational Neuroscience Branch, Intramural Research Program, National Institute of Mental Health, National Institute of Health, Bethesda, MD, USA; 48 Lieber Institute for Brain Development, Johns Hopkins University Medical Campus, Baltimore, MD, USA; 49 Department of Psychology, National University of Ireland, Galway, Ireland; 50 Department of Psychiatry, Neuropsychiatric Genetics Research Group, Trinity College Institute of Neuroscience, Trinity College Dublin, Dublin, Ireland; 51 Department of Psychiatry and Behavioral Sciences, Faculty of Medicine, University of Crete, Heraklion, Greece; 52 Helsinki Collegium for Advanced Studies, University of Helsinki, Helsinki, Finland; 53 Institute for Behavioral Genetics, University of Colorado, Boulder, CO, USA; 54 Institute of Clinical Medicine, University of Oslo, Oslo, Norway; 55 Department of Psychiatry, Hofstra Northwell School of Medicine, Hempstead, NY, USA

## Abstract

The complex nature of human cognition has resulted in cognitive genomics lagging behind many other fields in terms of gene discovery using genome-wide association study (GWAS) methods. In an attempt to overcome these barriers, the current study utilized GWAS meta-analysis to examine the association of common genetic variation (~8M single-nucleotide polymorphisms (SNP) with minor allele frequency ⩾1%) to general cognitive function in a sample of 35 298 healthy individuals of European ancestry across 24 cohorts in the Cognitive Genomics Consortium (COGENT). In addition, we utilized individual SNP lookups and polygenic score analyses to identify genetic overlap with other relevant neurobehavioral phenotypes. Our primary GWAS meta-analysis identified two novel SNP loci (top SNPs: rs76114856 in the *CENPO* gene on chromosome 2 and rs6669072 near *LOC105378853* on chromosome 1) associated with cognitive performance at the genome-wide significance level (*P*<5 × 10^−8^). Gene-based analysis identified an additional three Bonferroni-corrected significant loci at chromosomes 17q21.31, 17p13.1 and 1p13.3. Altogether, common variation across the genome resulted in a conservatively estimated SNP heritability of 21.5% (s.e.=0.01%) for general cognitive function. Integration with prior GWAS of cognitive performance and educational attainment yielded several additional significant loci. Finally, we found robust polygenic correlations between cognitive performance and educational attainment, several psychiatric disorders, birth length/weight and smoking behavior, as well as a novel genetic association to the personality trait of openness. These data provide new insight into the genetics of neurocognitive function with relevance to understanding the pathophysiology of neuropsychiatric illness.

## Introduction

Genome-wide association studies (GWAS) of complex quantitative phenotypes such as height^1^ and body mass index^2^ have successfully discovered and replicated hundreds of common variants meeting criteria for genome-wide significant association. By contrast, finding genetic loci associated with individual differences in cognitive ability using GWAS has proven challenging, despite considerable evidence from family and twin studies indicating that cognitive ability is highly heritable.^3^ For example, no genome-wide significant hits were detected in the earliest multi-cohort GWAS meta-analyses of general cognitive ability in ~3500 adults,^4^ or in ~5000 adults,^5^ or in ~18 000 youth.^6^ However, the first results attaining genome-wide significance, in three loci on chromosomes 6, 14 and 19, recently emerged in a GWAS meta-analysis of general cognitive function in 53  949 adults reported by the CHARGE Consortium.^7^ In addition, a recent study using data collected as part of the UK Biobank project reported three genomic regions significantly associated with performance on a test of verbal numerical reasoning (*N*=36 035), and two independent loci were significantly associated with performance on a reaction time task (*N*=111  483).^8^ However, in the same cohort, no genome-wide significant single-nucleotide polymorphisms (SNP)-based findings were detected for scores on a memory test, despite the large sample size (*N*=112  067), perhaps due to the low reliability of the very brief assay available.^8^

Recent GWAS meta-analyses of educational attainment, proposed as a proxy phenotype for cognition,^8, 9, 10, 11, 12^ have demonstrated that associations can be discovered with sufficient sample size, with the most recent analysis of 293  723 individuals yielding 74 independent SNPs that reached genome-wide significance.^9^ Nevertheless, this number of hits is an order of magnitude smaller than that reported for a similarly powered GWAS meta-analysis of height, which identified 697 variants that together explained ~16% of the variance for adult height in a sample of 253  288 individuals.^1^ Thus, the complex nature of human cognition, exacerbated by challenges of precise and reliable measurement, has rendered it a more difficult phenotype with which to gain traction in the era of GWAS discovery.

The importance of uncovering the molecular genetic basis of cognitive functioning is underscored by the fact that neurocognitive deficits represent a critical component of many neuropsychiatric disorders and disease states that can affect health outcomes across the lifespan. As examples, most early appearing neurodevelopmental disorders such as autism spectrum disorder^13^ and attention-deficit/hyperactivity disorder^14^ are associated with moderate to relatively impairing comorbid deficits in neurocognitive functioning. Longitudinally, lower cognitive ability scores in childhood have been linked to decreased rates of smoking cessation in adulthood.^15^ The major neuropsychiatric disorders that typically emerge in early adulthood, such as schizophrenia,^16^ bipolar disorder,^17^ anxiety disorders^18^ and depression,^19^ are also associated with a range of deficits in neurocognitive function. Human personality traits such as openness to new experiences^20^ and negative affect^21^ are, respectively, associated with better and worse neurocognitive performance. Individuals with debilitating neurological illnesses such as Parkinson's disease^22, 23^ and (by definition) the dementia spectrum including Alzheimer's disease^24^ also suffer from marked neurocognitive impairments. Further, early life cognitive performance can predict long-term development of illness,^25^ including mortality.^26^ From these findings, it has been suggested that general cognitive performance may index global bodily integrity, thereby permitting a potentially broad application of ‘cognitive epidemiology.'^27^

Deciphering the genetic overlap between cognition and risk for neuropsychiatric illness and other health-relevant traits can provide useful etiological insights and help prioritize likely causal relationships among complex human traits.^28^ Methods to estimate the genome-wide genetic correlation (*r*_g_) between two traits using summary GWAS statistics from published research studies utilizing linkage disequilibrium (LD) score regression procedures have recently become available.^28, 29^ LD score regression has been used recently to show significant genetic correlations between cognition-related phenotypes and cardiovascular disease,^27^ physical health^30^ and neuropsychiatric illness.^31^ However, the underlying causal variants and the genes through which they act have yet to be identified.^32^

There were two major aims of the current study: (1) conduct a large-scale (*n*=35 298) GWAS meta-analysis of general cognitive function in 24 independent cohorts, to identify SNP-based and gene-based loci associated with cognition; and (2) determine the extent of genetic correlation between general cognitive function and published neurobehavioral phenotypes of interest. These aims were executed within the context of the Cognitive Genomics Consortium (COGENT),^5, 10^ an international collaborative effort designed to study the molecular genetics of cognitive function.

## Materials and methods

### Participants

To date, COGENT has acquired individual-level neuropsychological, demographic, clinical and SNP array data from 24 studies (comprised of 35 sub-studies) enrolling 35  298 individuals (51.4% females, mean age of 45.6 (s.d.±8.6) years) of European ancestry drawn from the general population in North America, the United Kingdom and the European continent. Table 1 provides details of the individual study cohorts. A few of the cohorts overlap with those previously reported by the CHARGE consortium,^7^ so comparisons to the CHARGE report^7^ utilize 30 sub-studies comprising 27 888 fully independent subjects. All subjects provided written, informed consent to protocols approved by their institutional ethics boards in accordance with the Helsinki Declaration.

### General cognitive function phenotype

We examined general cognitive function (‘*g*'), a statistically derived broadband index of within-person performance on a neuropsychological test battery. The *g* phenotype estimates overall performance and is relatively invariant to the battery used and specific cognitive abilities assessed.^33, 34^ As in our prior reports,^5, 10^ for each cohort, *g* was determined using the first unrotated component extracted from a principal components analysis of individual test scores. Details on phenotypic assessments are provided in the supplement. Briefly, each COGENT sub-study (*n*=35) administered an average of 8 (s.d.±4) neuropsychological tests. To be included as a participant in COGENT, data from at least one neuropsychological measure across at least three domains of cognitive performance (for example, digit span for working memory; logical memory for verbal declarative memory; and digit symbol coding for processing speed), or the use of a validated *g*-sensitive measure was required. Digit symbol coding, digit span, verbal memory for words, visual memory, word reading, semantic fluency, verbal memory for stories, vocabulary, phonemic fluency and the trail-making test were the most common tests administered across cohorts. All individual test scores were adjusted (using multiple regression) for age^2^ and sex, as well as age × sex and age^2^ × sex interaction terms. The average internal consistency across test batteries was 71% (s.d.±12%), and the first unrotated principal component accounted for 42% (s.d.±11%) of the variance in overall test performance, which was expected based on an extensive prior literature (Supplementary Table S1).^35^

### Genotyping and imputation

All COGENT samples were genotyped on commercial Illumina or Affymetrix arrays, and a standardized GWAS quality control pipeline was developed and applied to the genetic data as described in detail in the Supplementary Information. Participants were of European ancestry, which was confirmed by analysis of genotype data using multidimensional scaling. Genetic clustering in each study was based on multidimensional scaling axis plotting versus four 1000 Genomes Project super populations (African, Admixed American, European and Asian), and non-European participants were removed. Genome-wide imputation was conducted using the largest available cosmopolitan reference cohort.^36^

### GWAS meta-analysis

In each COGENT sample, allelic association analysis of general cognitive function was conducted using imputed allele dosages and the first 10 principal components from the genotyped data to additionally adjust for population stratification. Cohorts of unrelated individuals (27 sub-cohorts) were analyzed using Plink 1.9.^37^ Samples including related individuals (8 sub-cohorts) were analyzed with BOLT-LMM^38^ using a mixed linear model association function that corrects for population stratification and relatedness.^39^ GWAS results were combined for meta-analysis of all 35 sub-studies using the inverse variance-weighted *Z*-score method in METAL.^40^ SNPs were filtered according to the following quality control thresholds: (1) minimum imputation quality INFO score of 0.60; (2) minor allele frequency at least 1% and (3) minimum 10 000 samples successfully imputed. Application of these filters resulted in a total of 8 037 763 high-quality SNPs available for meta-analysis in up to 35 298 samples. The standard threshold for genome-wide significance (*P*<5 × 10^−8^) was applied to SNP results of the GWAS meta-analysis.

### Gene-based analysis

Individual SNP results from the meta-analysis were aggregated to conduct a gene-based analysis using MAGMA.^41^ SNPs were mapped to genes based on NCBI build 37.3 and defined by the start and stop site ±5 kb, resulting in 18 164 autosomal genes. A genome-wide significance threshold for gene-based associations was calculated using the Bonferroni method (*α*=0.05/18 164; *P*=2.75 × 10^−6^).

### Genetic correlation analysis

LD score regression^28, 29^ was used to derive genetic correlations among GWAS results for general cognitive function and publicly available GWAS results from multiple neurobehavioral phenotypes of potential relevance. LD score regression quantifies the extent to which two phenotypes share genetic etiology (at least with respect to common variation captured by GWAS). GWAS summary statistics for 29 phenotypes were downloaded and processed similar to the pipeline of Bulik-Sullivan *et al.*^29^ The following phenotypes were included—cognition: childhood intelligence, educational attainment and obtaining a college degree; neurodevelopmental: autism and attention-deficit/hyperactivity disorder; neuropsychiatric: schizophrenia, bipolar disorder, anxiety and major depression; tobacco use: ever smoked cigarettes, number of cigarettes per day, age of onset of smoking and being a former smoker; personality: neuroticism, extraversion, openness, agreeableness and conscientiousness; brain volume: intracranial volume, nucleus accumbens, caudate nucleus, putamen, globus pallidus, hippocampus and thalamus; early childhood growth and development: infant head circumference, birth length and birth weight. URLs and references for data sources are provided in the Supplementary Information.

## Results

### GWAS of general cognitive function

**Figure 1:**
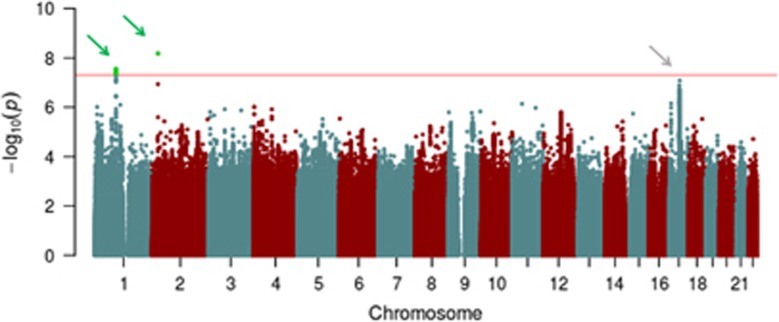
Manhattan plot depicting results of genome-wide association study meta-analysis for general cognitive function. Green arrows indicate loci attaining genome-wide significance (red line, *P*<5 × 10^−8^). Gray arrow indicates locus at chromosome 17q21.31 approaching genome-wide significance.

**Figure 2:**
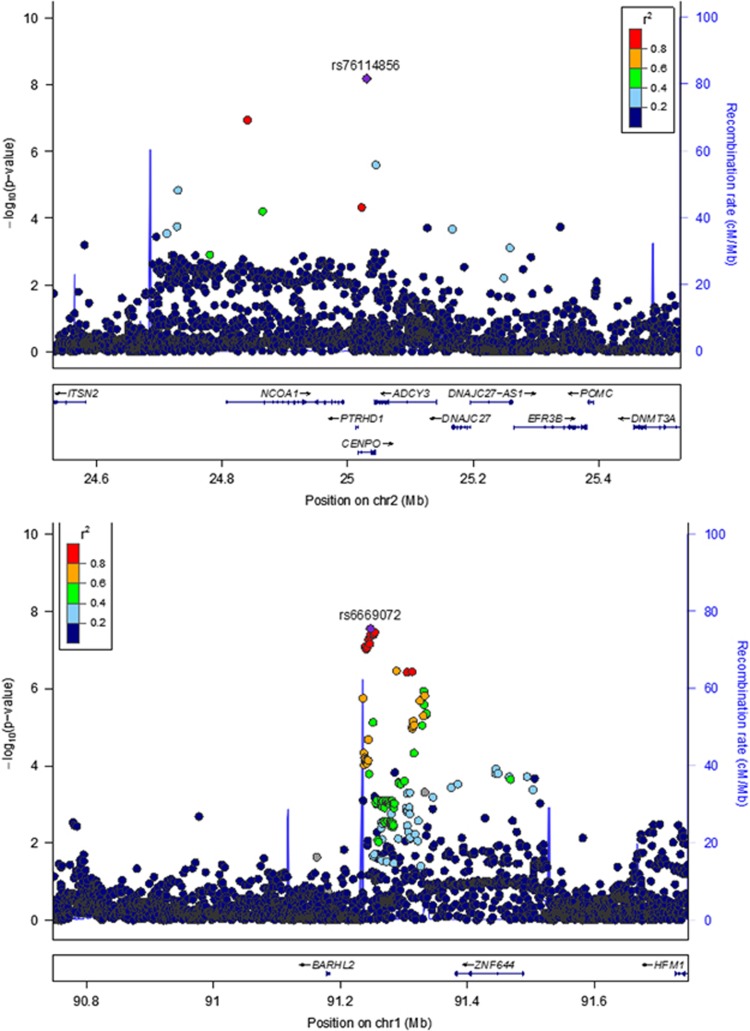
Region plots depicting genome-wide significant loci on Chromosome 2 (top) and Chromosome 1 (bottom). Local linkage disequilibrium (*r*^2^) is color-coded as shown in the legend, and local recombination rate is depicted by the bright blue peaks (magnitude indicated by the right-hand *y* axis).

The QQ plot (Supplementary Figure 1) demonstrates *λ*_GC_ was 1.12, comparable to the value (1.14) observed in the recent CHARGE meta-analysis of cognitive ability.^7^ The LD score regression intercept of 1.04 indicates that polygenicity, rather than residual population stratification, accounted for most of the increase in the mean *χ*^2^ statistic.^28, 29^ As shown in the Manhattan plot (Figure 1), two loci surpassed the genome-wide threshold of *P*⩽5 × 10^−8^ in our GWAS meta-analysis (see Supplementary Table S2 for more details). On chromosome 2 (Figure 2, top), intronic SNP rs76114856 in the centromere protein O (*CENPO)* gene was genome-wide significant (*P*=6.58 × 10^−9^). On chromosome 1 (Figure 2, bottom), a cluster of six SNPs located in a lincRNA, RP4-665J23.1 (also known as *LOC105378853*), were also genome-wide significant (top SNP, rs6669072, *P*=2.77 × 10^−8^). Values of meta-analytic tests of heterogeneity were low and not statistically significant, indicating that outlier cohorts did not drive significant results. In addition, a large 1.4 Mb region at chromosome 17q21.31, coextensive with a known inversion polymorphism,^42^ harbored 101 nearly significant SNPs (all *P*'s<10^−6^; top SNP, rs916888, *P*=8.18 × 10^−8^).

### Gene-based analysis of general cognitive function

Seven genes in three chromosomal regions (*WNT3*, *PLEKHM1* and *ARHGAP27* at chromosome 17q21.31; *TP53* and *WRAP53* at chromosome 17p13.1; and *ATXN7L2* and *CYB561D1* at chromosome 1p13.3) were significantly associated with cognitive function after Bonferroni correction (Table 2). Several genes at these loci, including *NSF*, *STH*, *KANSL1*, *CRHR1* and *MAPT* in the 17q21.31 inversion region, demonstrate association just below the Bonferroni-corrected threshold.

### SNP lookups from published GWAS of cognition and educational attainment

We sought to expand the utility of our data by using it as a lookup table to confirm and extend associations previously reported in large-scale GWAS of cognition (from the CHARGE consortium^7^ and the UK Biobank^8^) and educational attainment (from the SSGAC consortium^9^). First, we looked up all cognitive SNPs nominally associated in the CHARGE study at *P*<10^−5^. Importantly, because of partial sample overlap between COGENT and CHARGE, we re-ran our cognitive GWAS excluding five overlapping cohorts (CHS, FHS, HBCS, LBC1936 and NCNG). A meta-analytic *P*-value was then generated across the two studies for those loci with nominal *P*<0.05 in COGENT. As shown in Supplementary Table S3, we found support for the three genome-wide significant loci reported in CHARGE, as well as support for an additional, novel locus at chromosome 3p22.3 that attained a meta-analytic *P*-value surpassing the genome-wide significance threshold (rs1523041, *P*=5.46 × 10^−10^). This SNP is intergenic; however, publicly available gene expression data (from GTEx^43^) have shown that rs1523041 is an expression quantitative trait locus for the *ARPP21* gene (Supplementary Figure 1).

Next, we examined the genome-wide significant SNPs reported from the UK Biobank study of verbal numerical reasoning and reaction time to determine if these were also associated with general cognitive ability. As shown in Supplementary Table S4, we found nominally significant support for the chromosome 22 locus associated with verbal numerical reasoning (top local SNP in COGENT, rs12170228, *P*=0.0053, same direction of effect in COGENT and UK Biobank). We also showed a similar trend for the chromosome 7 locus associated with verbal numerical reasoning (rs9771228, *P*=0.074 in COGENT, same direction of effect in COGENT and UK Biobank). The lone SNP on chromosome 14 that attained genome-wide significance in the UK Biobank GWAS of verbal numerical reasoning was a rare variant (MAF=0.1%) that was not available in COGENT and has no known proxies. For the two loci reported to be associated with reaction time in UK Biobank, COGENT results demonstrated the same direction of allelic effects, but were not statistically significant (COGENT *P*'s>0.6).

Finally, we looked up all SNPs that represented independent, genome-wide significant hits for educational attainment from the combined SSGAC+UK Biobank cohorts. Of a total of 164 SNPs meeting this criteria (as listed in Table 1.16 of that report^9^), 143 SNPs were directly available in COGENT, and an additional 12 SNPs were tested by proxy (9 SNPs were unavailable in COGENT even by proxy). There were 31 educational attainment SNPs that were nominally significant at *P*<0.05 in COGENT, all in the same direction of effect as for educational attainment, representing a highly significant enrichment (*P*=3.9 × 10^−11^, binomial test) of overlap between years of education and cognitive function (Supplementary Table S5). Further, two SNPs (rs7593947 and rs2568955) were Bonferroni-corrected significant (for 155 tests; *P*<3.23 × 10^−4^), suggesting two specific loci for cognition were discovered using this ‘proxy-phenotype' method.^11^ Notably, GTEx data reveal that rs2568955 is a strongly significant expression quantitative trait locus (Supplementary Figure 2) in brain tissue for *RPL31P12*, although this gene is annotated as a pseudogene; rs7593947 is an intronic variant in the *BCL11A* gene.

### Genetic correlation of general cognitive function with related complex traits

SNP heritability (due to common variation) as calculated using LD score regression was 21.5% (s.e.=0.01%). This value for *h*^2^_g_ is slightly lower than prior studies^7, 8^ which utilized the GCTA approach in single samples; this attenuation is expected based on the fact that LD score regression utilizes summary scores as opposed to full SNP data. The results of the LD score regression-based genetic correlations with other neurobehavioral phenotypes are detailed in Table 3. Note that our use of the LD score regression approach applied a stringent correction (unconstrained intercept) for potential sample overlap and population stratification, as well as a conservative Bonferroni correction for multiple phenotypes. Not surprisingly, strongly significant positive correlations were observed with the most closely related phenotypes: years of education (*P*=1.48 × 10^−63^), obtaining a college degree (*P*=1.88 × 10^−23^) and childhood intelligence (*P*=1.24 × 10^−13^). A significant negative correlation was observed with schizophrenia (*P*=4.09 × 10^−4^), such that genetic load for lower cognitive scores was associated with greater risk for schizophrenia, consistent with prior reports from COGENT^5^ and others.^28, 30^ Similar trends were observed for attention-deficit/hyperactivity disorder and anxiety, with nominal (but not Bonferroni-corrected) levels of statistical significance. A Bonferroni-corrected significant positive correlation was observed for autism (*P*=6.00 × 10^−4^), again consistent with prior reports.^30, 44^

A novel observation is a significant, positive genetic correlation between general cognitive ability and the personality trait of openness (*r*_g_=0.48; *P*=3.25 × 10^−4^); no other correlations with personality traits were even nominally significant. (It should be noted that agreeableness was the only trait for which the SNP-based heritability did not significantly differ from zero (*h*^2^_g_=0.016; s.e.=0.029) and was, therefore, not included in correlational analyses). Higher cognitive ability was strongly genetically correlated with reduced rates of smoking (*P*=2.13 × 10^−4^) and greater rates of quitting smoking (*P*=5.09 × 10^−3^).

Nominally significant positive genetic correlations were observed with birth length and weight, with similar trends for infant head circumference. All genetic correlations with neuroanatomic measures trended in the positive direction (larger brain volumes associated with higher cognitive ability), consistent with a large literature revealing correlations at the phenotypic level,^45^ although none of the genetic correlations attained nominal significance under our conservative approach.

## Discussion

Our GWAS meta-analysis of general cognitive function in a sample of 35 298 individuals of European ancestry revealed two novel associated SNP loci, three novel gene-based loci, and provided added support for several previously reported associations. Strengths of our study included access to individual-level genetic and neuropsychological data, which allowed us to run each sample through uniform genotype and phenotype quality control pipelines. Specifically, the general cognitive function phenotype was well characterized as a composite score derived from relatively large batteries of both verbal and nonverbal neuropsychological tests. Genotype data were processed with the latest imputation platforms and analytic procedures.

Our top GWAS hit was rs76114856, of which the minor T allele was associated with reduced cognitive performance. This SNP is an intronic variant in the *CENPO* gene, which encodes a component of the interphase centromere complex.^46^ This gene is highly expressed in the basal ganglia and thalamus of the human brain.^65^ *CENPO* is located at chromosome 2p23.3 and has prior GWAS associations to height.^1, 47^ The *CENPO* gene also had a nominal association to cognition in our gene-based analysis at *P*<0.05, as did neighboring genes *NCOA1*, *PTRHD1* and *ADCY3*. The second strongest GWAS signal fell within a large intergenic non-coding RNA (lincRNA) of unknown function, RP4-665J23.1. Neighboring protein-coding genes are poorly annotated and do not provide strong clues as to the potential biological mechanism underlying the association.

We also found evidence that the chromosome 17q21.31 inversion region is associated with cognitive function. The chromosome 17q21.31 inversion consists of two haplotypes (H1 and H2), and the absence of recombination across the ~1.5 Mb region between the inverted (H2) and the noninverted (H1) chromosomes has resulted in two families of chromosomes.^48^ H1 chromosomes comprise the common (~80% frequency in European samples) noninverted gene order, whereas the H2 haplotype comprises the inverted gene order (~20% in European samples).^48^ There are several sources of evidence that variation at this locus is associated with neurobehavioral phenotypes. For example, the 17q21.31 microdeletion syndrome is associated with the H2 haplotype, which carries additional low-copy repeats susceptible to non-allelic homologous recombination. The syndrome is characterized clinically by developmental delay/intellectual disability, neonatal/childhood hypotonia, friendly behavior and specific facial dysmorphisms.^49^ Notably, *KANSL1* gene disruption is associated with the full clinical spectrum of 17q21.31 microdeletion syndrome.^49^ In addition, the region harbors the *MAPT* gene, encoding microtubule-associated protein tau, a hallmark of multiple dementias.^48, 50, 51, 52^ The H1 family of haplotypes has been associated with increased risk for late-life tauopathies, diseases marked by the accumulation of MAPT neurofibrillary tangles in nerve cells, such as sporadic frontotemporal dementia,^53^ Alzheimer's disease,^54^ Parkinson's disease^55^ and progressive supranuclear palsy.^48^ By contrast, the H2 haplotype has been associated with developmental delay and learning difficulties,^51, 56, 57, 58^ as well as reduced intracranial volume.^52^ Consistent with these latter observations, our data suggest alleles corresponding to the H2 haplotype are associated with worse cognitive performance.

In addition to the loci attaining clear genome-wide significance through our primary SNP-based and gene-based analyses, our results confirmed and extended prior GWAS studies of cognitive and educational phenotypes. Although a prior COGENT report provided converging evidence for a role of a chromosome 6 locus (rs1906252),^10^ we now provide further support for the *NPAS3*/*AKAP6* locus on chromosome 14 previously reported by the CHARGE consortium. *NPAS3* is a promising candidate gene, as it has a role in neurodevelopment, and disruptions of this gene have been associated with psychiatric and intellectual disability phenotypes.^59, 60^

In the context of prior associations to cognitive and educational phenotypes, our data identified several loci with strong empirical support for a role in cognition. Of these, two are noteworthy for representing known expression quantitative trait locus, permitting inference of potential biological mechanisms underlying the statistical associations. Specifically, we found that the major (C) allele at rs1523041 was strongly (*P*=5.46 × 10^−10^) associated with better cognitive performance; this allele drives lower expression of the *ARPP21* gene (Supplementary Figure S1). *ARPP21* encodes a cAMP-regulated phosphoprotein, enriched in the basal ganglia and cerebellum, that has a central role in the integration of neurotransmitter inputs into striatal medium spiny neurons.^61^ Intriguingly, a deletion encompassing this gene segregated with syndromic intellectual disability in a multiply affected pedigree.^62^ Similarly, we found that the minor (T) allele of rs2568955 was associated with poorer cognitive performance, and this allele is associated with greater expression of *RPL31P12* (Supplementary Figure S2). It should be noted that the strongest expression quantitative trait locus associations for these SNPs were observed in non-brain tissue in the GTEx database, perhaps due to smaller sample sizes available for neuronal phenotypes; these results should be tested in larger studies of brain expression that will soon be forthcoming. *BCL11A* is also a promising candidate gene for cognition. Haploinsufficiency of this gene has been associated with intellectual disability in a large clinical study, with the phenotype recapitulated in *Bcl11a* knockout mice, which was shown to be mediated through downstream transcriptional dysregulation in the hippocampus and cortex.^63^

Analysis of the genetic correlation between general cognitive function and various other phenotypes revealed that better cognitive performance was robustly genetically correlated with more years of schooling, decreased likelihood of smoking and decreased risk for several psychiatric disorders (as well as increased risk for autism). These results are generally consistent with recent genetic correlation studies of cognitive phenotypes^30^ and proxy phenotypes for cognition.^9^ The personality trait of openness, a core component of the ‘Big 5' model of personality, was positively correlated with cognitive ability at the genetic level. This novel finding is consistent with a prior literature in which moderate phenotypic correlations (values for *r* ranging between 0.25 and 0.5) between openness and cognitive ability have been repeatedly observed,^20, 64^ whereas cognition is generally uncorrelated with other personality dimensions. Moreover, phenotypic data from twin and family studies have suggested a specific genetic correlation between openness and general cognitive ability.^65^ Longitudinal studies have suggested a model in which openness may serve as a ‘buffer' against cognitive decline, as has been proposed in the Openness-Fluid-Crystallized-Intelligence model applied to late adulthood.^66^ Positive genetic correlations with birth length and weight suggest a critical role for prenatal developmental factors in the subsequent manifestation of cognitive ability throughout the lifespan.

One limitation of the current study is the wide age range of subjects, both across cohorts and within cohorts. Although we sought to control for confounding effects of age using covariates, genetic influence on cognitive ability is somewhat reduced in early childhood and adolescence relative to adulthood.^67^ In addition, late-life effects of cognitive decline may be mediated through somewhat disparate molecular pathways; this may explain the relatively weak effect of variation at *APOE* compared with prior GWAS meta-analysis.^7^ Nevertheless, cognitive abilities are remarkably stable across the entire lifespan,^68^ so we chose to include all available samples in order to maximize sample size and power.

Similarly, we chose to include cohorts with widely disparate neurocognitive batteries, which undoubtedly contributed to noise surrounding the estimates of *g.* Moreover, as demonstrated in Supplementary Table 1, the degree to which the first principal component captured the shared variance across tests was heterogeneous across cohorts. In general, cohorts with fewer available tests demonstrated greater loading onto the first factor, but with less reliability as determined by Cronbach's *α*. However, in each case, the scree plots clearly demonstrated a steep drop in variance accounted for beyond the first component, consistent with the known properties of *g.* Moreover, in a subset of subjects in one of the cohorts (TOP), we previously^5^ compared our computed *g* with estimated intelligence quotient from a 4-subscale composite from the WASI,^69^ and observed a strong correlation (*r*=0.67, *P*<10^−46^). Thus, we are confident that our computed index for each cohort was primarily reflecting general cognitive ability, but it is equally certain that substantial heterogeneity existed across cohorts, thereby reducing power in comparison with more easily measured quantitative traits such as height. Notably, it has been empirically demonstrated that such noise is more than compensated by increases in statistical power.^70^ Given the expense in conducting comprehensive cognitive assessments, we chose to include all available cohorts meeting our basic criteria.

Despite the statistical significance of the novel GWAS loci identified in the current report, it is important to emphasize that the effect sizes for individual SNPs are very small; each of our top two SNPs individually account for ~0.1% of the variance in cognitive performance. For context, these effect sizes are considerably smaller than those observed for the top individual loci associated with other quantitative anthropometric traits such as height and weight,^1, 2^ This difference may reflect the complexity of the underlying genetic architecture of cognition, as >80% of all genes are expressed in brain;^71^ this complexity has also slowed progress in identifying genetic loci for neuropsychiatric disorders, given the potentially large mutational target.^70^ This challenge is exacerbated by the fact that general cognitive ability is a latent trait that is only indirectly captured by the available phenotypic measures, which are also quite heterogeneous across cohorts. Moreover, the well-known winners' curse phenomenon^72^ will likely result in even further reduction of our effect size estimates in future studies of independent cohorts. However, as described in the preceding paragraphs, results of the present study can provide important information about the molecular underpinnings of cognitive function as well as clues relevant to the etiology neuropsychiatric disorders and other conditions relevant to human health. Although it is striking that general cognitive ability remains the quantitative trait most challenging to GWAS methodology, the recent success of very large-scale GWAS in educational attainment^9^ provides optimism that cognition is now at the beginning of the slope of increasing GWAS discovery that has been observed for all heritable complex traits.^73^

## Figures and Tables

**Table 1:** Demographic characteristics of the consortium

*Cohort*	*Study name*	*Country*	N	*Age mean*	*Age s.d.*	*Min age*	*Max age*	N *Male*	*% Male*
ACPRC	Age and Cognitive Performance Research Cohort	UK	1461	64.7	6.1	47	85	425	0.29
ADNI	Alzheimer's Disease Neuroimaging Initiative	USA	259	75.3	5.1	62	90	137	0.53
ASPIS	Athens Study of Psychosis Proneness and Incidence of Schizophrenia	Greece	919	20.7	1.9	18	25	919	1.00
CAMH	Center for Addiction and Mental Health	Canada	80	48.6	19.4	18	86	38	0.48
CHS	Cardiovascular Health Study	USA	2931	77.3	5.3	69	96	1208	0.41
CNP	UCLA Consortium for Neuropsychiatric Phenomics	USA	628	31.1	8.3	21	50	310	0.49
DCC	Duke Cognition Cohort	USA	1193	27.1	11.6	18	77	558	0.47
DNS	Duke Neurogenetics Study	USA	455	19.8	1.3	18	22	212	0.47
DUBLIN	Galway and Dublin, Ireland	Ireland	135	36.2	12.5	18	60	71	0.53
FHS	Framingham Heart Study	USA	5360	51.7	10.8	25	87	2460	0.46
GCAP	NIMH Genes, Cognition and Psychosis Program	USA	964	33.8	9.8	18	61	438	0.45
GENADA	Genotype–Phenotype Associations in Alzheimer's Disease	Canada	767	73.4	7.9	48	94	279	0.36
HBCS	Helsinki Birth Cohort Study	Finland	299	67.7	2.3	64	75	299	1.00
IBG	Institute for Behavioral Genetics	USA	260	15.9	1.5	12	19	235	0.90
LBC1936	Lothian Birth Cohort 1936 Study	UK	951	69.6	0.8	68	71	509	0.54
LLFS	Long Life Family Study	USA and Denmark	4081	68.1	14.0	24	90	1861	0.46
LOAD	Late Onset Alzheimer's Disease Family Study	USA	1033	75.1	5.7	53	95	379	0.37
LOGOS	Learning on Genetics of Schizophrenia Spectrum	Crete	795	22.5	3.8	18	37	795	1.00
MCTFR	Minnesota Center for Twin and Family Research	USA	5448	32.5	14.2	17	65	2349	0.43
MUNICH	Munich, Germany	Germany	1095	47.8	15.3	19	76	540	0.49
NCNG	Norwegian Cognitive NeuroGenetics Study	Norway	625	47.6	18.3	18	79	214	0.34
PNC	Philadelphia Neurodevelopmental Cohort	USA	4711	13.8	3.6	8	21	2440	0.52
TOP	Thematic Organized Psychosis Research Study	Norway	661	31.9	8.9	16	55	359	0.54
ZHH	Zucker Hillside Hospital	USA	187	35.2	18.8	8	78	108	0.58

**Table 2:** Results of gene analysis (top 20 genes)

*Symbol*	*Gene name*	*Chr*	*Start*	*Stop*	*SNPs*	*Parameters*	N	Z *stat*	P*-value*
***WNT3***	**Wingless-type MMTV integration site family, member 3**	**17q21.31**	**44836686**	**44901082**	**127**	**46**	**29 063**	**5.4753**	**2.18E−08**
***PLEKHM1***	**Pleckstrin homology and RUN domain-containing M1**	**17q21.31**	**43508266**	**43573146**	**123**	**23**	**27 885**	**4.9724**	**3.31E−07**
***TP53***	**Tumor protein p53**	**17p13.1**	**7566720**	**7595863**	**94**	**32**	**31 813**	**4.9082**	**4.60E−07**
***ARHGAP27***	**Rho GTPase-activating protein 27**	**17q21.31**	**43466268**	**43515282**	**128**	**17**	**25 183**	**4.8923**	**4.98E−07**
***CYB561D1***	**Cytochrome b561 family, member D1**	**1p13.3**	**110031658**	**110048063**	**27**	**12**	**31 670**	**4.7687**	**9.27E−07**
***WRAP53***	**WD repeat containing, antisense to TP53**	**17p13.1**	**7584389**	**7611820**	**63**	**20**	**32 133**	**4.6221**	**1.90E−06**
***ATXN7L2***	**Ataxin 7-like 2**	**1p13.3**	**110021561**	**110040426**	**29**	**11**	**33 153**	**4.5842**	**2.28E−06**
*PSMA5*	Proteasome subunit alpha 5	1p13.3	109936653	109974108	62	23	32 881	4.4835	3.67E−06
*NSF*	N-ethylmaleimide sensitive factor	17q21.31	44663035	44839830	89	20	29 574	4.4412	4.47E−06
*SORT1*	Sortilin 1	1p13.3	109847187	109945563	147	36	33 175	4.4403	4.49E−06
*SYPL2*	Synaptophysin-like 2	1p13.3	110004100	110029764	66	16	34 387	4.4171	5.00E−06
*CFAP99*	Cilia- and flagella-associated protein 99	4p16.3	2415701	2469690	171	43	33 195	4.3569	6.60E−06
*KANSL1*	KAT8 regulatory NSL complex subunit 1	17q21.31	44102282	44307740	775	22	27 788	4.3536	6.70E−06
*STH*	Saitohin	17q21.31	44071616	44082060	56	12	26 909	4.3028	8.43E−06
*SPPL2C*	Signal peptide peptidase-like 2C	17q21.31	43917256	43929438	69	8	30 906	4.2974	8.64E−06
*SP140L*	SP140 nuclear body protein like	2q37.1	231186894	231273445	365	51	34 250	4.1532	1.64E−05
*LRRC37A*	Leucine-rich repeat containing 37A	17q21.31	44367497	44420160	5	3	26 694	4.1414	1.73E−05
*MAPT*	Microtubule-associated protein tau	17q21.31	43966748	44110700	725	33	29 040	4.079	2.26E−05
*CRHR1*	Corticotropin-releasing hormone receptor 1	17q21.31	43692710	43918194	1024	75	30 191	3.7486	8.89E−05
*PCDH15*	Protocadherin-related 15	10q21.1	55557531	56566051	4082	531	33 480	3.218	6.46E−04

Abbreviation: SNP, single-nucleotide polymorphism. Genes that are in bold font were significant after genome-wide Bonferroni correction.

**Table 3:** Results of genetic correlation using LD score regression

*Genetic correlation with other traits using LD score regression*					
*Group*	*Phenotype*	r_*g*_	*s.e.*	z	P*-value*
Cognition	***Childhood IQ***	***0.89***	***0.12***	***7.41***	***1.24E*****−*****13***
	***College degree***	***0.66***	***0.07***	***9.98***	***1.88E*****−*****23***
	***Years of education***	***0.73***	***0.04***	***16.83***	***1.48E*****−*****63***
Neuropsychiatric	**ADHD**	**−0.35**	**0.16**	**−2.14**	**3.22E−02**
	Alzheimer's	**−**0.13	0.11	**−**1.17	2.41E**−**01
	Anorexia	**−**0.02	0.10	**−**0.24	8.08E**−**01
	**Anxiety**	**−0.50**	**0.19**	**−2.57**	**1.03E−02**
	***Autism***	***0.28***	***0.08***	***3.43***	***6.00E*****−*****04***
	Bipolar	0.00	0.08	**−**0.06	9.52E**−**01
	Major depression	0.10	0.10	0.96	3.35E**−**01
	***Schizophrenia***	**−*****0.17***	***0.05***	**−*****3.53***	***4.09E*****−*****04***
Personality	Extraversion	**−**0.13	0.10	**−**1.36	1.74E**−**01
	Agreeableness	1.24	1.24	1.00	3.17E**−**01
	Conscietousness	0.10	0.14	0.74	4.61E**−**01
	***Openness***	***0.48***	***0.13***	***3.59***	***3.25E*****−*****04***
	Neuroticism	**−**0.18	0.12	**−**1.49	1.35E**−**01
Smoking	Age of onset	0.21	0.13	1.67	9.49E**−**02
	Cigarettes per day	0.03	0.11	0.27	7.85E**−**01
	**Ever smoker**	**−0.24**	**0.08**	**−3.07**	**2.13E−03**
	**Former smoker**	**0.29**	**0.10**	**2.80**	**5.09E−03**
Brain volume	Accumbens	0.26	0.15	1.74	8.18E**−**02
	Caudate	0.08	0.10	0.79	4.30E**−**01
	Hippocampus	0.24	0.13	1.88	6.06E**−**02
	Intracranial volume	0.14	0.13	1.09	2.77E**−**01
	Pallidum	0.16	0.13	1.28	2.02E**−**01
	Putamen	0.13	0.09	1.44	1.50E**−**01
	Thalamus	0.13	0.12	1.07	2.83E**−**01
Early growth	**Birth length**	**0.20**	**0.09**	**2.13**	**3.33E−02**
	**Birth weight**	**0.15**	**0.05**	**2.89**	**3.90E−03**
	Infant head Circumference	0.19	0.11	1.69	9.04E**−**02

Abbreviations: ADHD, attention-deficit/hyperactivity disorder; IQ, intelligence quotient; LD, linkage disequilibrium. Traits that are in bold font were nominally significant (*P*<0.05); traits that are italicized were significant after Bonferroni correction.
