# Erratum: GWAS meta-analysis reveals novel loci and genetic correlates for general cognitive function: a report from the COGENT consortium

**DOI:** 10.1038/mp.2017.197

**Published:** 2017-09-12

**Authors:** J W Trampush, M L Z Yang, J Yu, E Knowles, G Davies, D C Liewald, J M Starr, S Djurovic, I Melle, K Sundet, A Christoforou, I Reinvang, P DeRosse, A J Lundervold, V M Steen, T Espeseth, K Räikkönen, E Widen, A Palotie, J G Eriksson, I Giegling, B Konte, P Roussos, S Giakoumaki, K E Burdick, A Payton, W Ollier, M Horan, O Chiba-Falek, D K Attix, A C Need, E T Cirulli, A N Voineskos, N C Stefanis, D Avramopoulos, A Hatzimanolis, D E Arking, N Smyrnis, R M Bilder, N A Freimer, T D Cannon, E London, R A Poldrack, F W Sabb, E Congdon, E D Conley, M A Scult, D Dickinson, R E Straub, G Donohoe, D Morris, A Corvin, M Gill, A R Hariri, D R Weinberger, N Pendleton, P Bitsios, D Rujescu, J Lahti, S Le Hellard, M C Keller, O A Andreassen, I J Deary, D C Glahn, A K Malhotra, T Lencz

**Keywords:** Genome-wide association studies, Genetics of the nervous system, Cognitive neuroscience

**Correction to:** *Molecular Psychiatry* (2017) **22**, 336–345; doi:10.1038/mp.2016.244published online 17 January 2017

Data access for several cohorts used in this study was provided by the National Center for Biotechnology Information (NCBI) database of Genotypes and Phenotypes (dbGaP). dbGaP accession numbers for these cohorts were as follows:

Cardiovascular Health Study (CHS): phs000287.v4.p1, phs000377.v5.p1 and phs000226.v3.p1.

Framingham Heart Study (FHS): phs000007.v23.p8 and phs000342.v11.p8.

Multi-Site Collaborative Study for Genotype-Phenotype Associations in Alzheimer’s Disease (GENADA): phs000219.v1.p1.

Long Life Family Study (LLFS): phs000397.v1.p1.

Genetics of Late Onset Alzheimer’s Disease Study (LOAD): phs000168.v1.p1.

Minnesota Center for Twin and Family Research (MCTFR): phs000620.v1.p1.

Philadelphia Neurodevelopmental Cohort (PNC): phs000607.v1.p1.

The acknowledgment statements for these cohorts are provided below.

Framingham Heart Study: The Framingham Heart Study is conducted and supported by the National Heart, Lung, and Blood Institute (NHLBI) in collaboration with Boston University (contracts N01HC25195 and HHSN268201500001I). This article was not prepared in collaboration with investigators of the Framingham Heart Study and does not necessarily reflect the opinions or views of the Framingham Heart Study, Boston University, or the NHLBI. Funding for SHARe Affymetrix genotyping was provided by NHLBI contract N02HL64278. SHARe Illumina genotyping was provided under an agreement between Illumina and Boston University.

Cardiovascular Health Study (CHS): this research was supported by contracts HHSN268201200036C, HHSN268200800007C, N01HC85079, N01HC85080, N01HC85081, N01HC85082, N01HC85083, N01HC85084, N01HC85085, N01HC85086, N01HC35129, N01HC15103, N01HC55222, N01HC75150, N01HC45133 and N01HC85239; grants U01 HL080295 and U01 HL130014 from the NHLBI, and R01AG023629 from the National Institute on Aging, with additional contribution from the National Institute of Neurological Disorders and Stroke. A full list of principal CHS investigators and institutions can be found at https://chs-nhlbi.org/pi. This article was not prepared in collaboration with CHS investigators and does not necessarily reflect the opinions or views of CHS or the NHLBI. Support for the genotyping through the CARe Study was provided by NHLBI contract N01HC65226. Support for the CHS Whole Genome Study was provided by NHLBI grant HL087652. Additional support for infrastructure was provided by HL105756, and additional genotyping among the African-American cohort was supported in part by HL085251; DNA handling and genotyping at Cedars-Sinai Medical Center were supported in part by National Center for Research Resources grant UL1RR033176, now through National Center for Advancing Translational Technologies CTSI grant UL1TR000124; in addition to National Institute of Diabetes and Digestive and Kidney Diseases grant DK063491 to the Southern California Diabetes Endocrinology Research Center.

Multi-Site Collaborative Study for Genotype-Phenotype Associations in Alzheimer’s Disease: the genotypic and associated phenotypic data used in the study were provided by the GlaxoSmithKline, R&D Limited. Details on data acquisition were published previously (Li H, Wetten S, Li L, St Jean PL, Upmanyu R, Surh L *et al.*, Candidate single-nucleotide polymorphisms from a genome-wide association study of Alzheimer disease, *Arch Neurol* 2008; **65**: 45–53 and Filippini N, Rao A, Wetten S, Gibson RA, Borrie M, Guzman D *et al.*, Anatomically-distinct genetic associations of APOE epsilon4 allele load with regional cortical atrophy in Alzheimer’s disease, *Neuroimage* 2009; **44**: 724–728).

Genetics of Late Onset Alzheimer’s Disease Study: funding support for the Genetic Consortium for Late Onset Alzheimer’s Disease was provided through the Division of Neuroscience, National Institute on Aging (NIA). The consortium includes a genome-wide association study funded as part of the Division of Neuroscience, NIA. Assistance with phenotype harmonization and genotype cleaning, as well as with general study coordination, was provided by the consortium. A list of contributing investigators is available at https://www.ncbi.nlm.nih.gov/projects/gap/cgi-bin/study.cgi?study_id=phs000168.v1.p1.

Long Life Family Study: funding support for the Long Life Family Study was provided by the Division of Geriatrics and Clinical Gerontology, NIA. The study includes GWAS analyses for factors that contribute to long and healthy life. Assistance with phenotype harmonization and genotype cleaning as well as with general study coordination was provided by the Division of Geriatrics and Clinical Gerontology, NIA. Support for the collection of data sets and samples was provided via Multicenter Cooperative Agreement support by the Division of Geriatrics and Clinical Gerontology, NIA (UO1AG023746, UO1023755, UO1023749, UO1023744 and UO1023712). Funding support for the genotyping, which was performed at the Johns Hopkins University Center for Inherited Disease Research, was provided by the NIA.

Minnesota Center for Twin and Family Research: this project was led by William G. Iacono, PhD and Matthew K. McGue, PhD (co-principal investigators) at the University of Minnesota. Co-investigators from the same institution included Irene J. Elkins, Margaret A. Keyes, Lisa N. Legrand, Stephen M. Malone, William S. Oetting, Michael B. Miller and Saonli Basu. Funding support for this project was provided through the National Institute on Drug Abuse (U01DA024417). Other support for sample ascertainment and data collection came from grants R37DA05147, R01AA09367, R01AA11886, R01DA13240 and R01MH66140.

Philadelphia Neurodevelopmental Cohort: support for the collection of the data sets was provided by grant RC2MH089983 to Raquel Gur, MD and RC2MH089924 to Hakon Hakonarson, MD, PhD. All subjects were recruited through the Center for Applied Genomics at The Children’s Hospital in Philadelphia.

