# Supplementary material for: GWAS meta-analysis reveals novel loci and genetic correlates for general cognitive function: a report from the COGENT consortium
Source: Mol Psychiatry. 2017 Jan 17;22(3):336–45. doi: 10.1038/mp.2016.244 (PMC5322272; doi:10.1038/mp.2016.244)
Supplement: Supplementary Information [file mp2016244x1.docx]

**Supplemental Information**

**GWAS meta-analysis reveals novel loci and genetic correlates for general cognitive function: A report from the COGENT consortium**

**Contents**

**1. Supplemental Methods and Materials**

A. Individual Study Details

B. Neurocognitive Domains and Tests

C. Phenotyping Procedures

D. Genotyping Procedures

E. Statistical and Analytical Procedures

F. Sources of Data for LD Score Regression

**2. Supplemental Figures**

**3. Supplemental References**

**1. Supplemental Methods and Materials**

**A. Individual Study Details**

Below is a description of each individual study that was included in the current analysis. To the extent possible, details are provided regarding recruitment procedures, informed consent, sample demographics, and a brief description of the genome-wide genotyping procedures. Individual studies are listed alphabetically. Additional details of each study have been published and references to those studies provided.

**ACPRC (****Age and Cognitive Performance Research Cohort)**

The ACPRC Manchester and Newcastle Longitudinal Studies of Aging is a longitudinal research program in the UK.^1^ Volunteers were recruited by advertisements in local community centers, newspapers, radio, and television. These began in Manchester in 1984 to 1986, with refreshment samples recruited in 1989/90 and 1991/92. The only exclusion criteria were that volunteers were at least age 45 years and were able to attend a regional test centre independently. The study was funded by the U.K. Social Science Research Council from 1983 to 1988, then jointly by the U.K. Medical Research Council and the Economic and Social Research Council (ESRC) from 1988 to 1993 and then by the Medical Research Council (MRC) from 1993 to 1998. From 1998 to 2003 the study continued with support from ESRC, the Wellcome Trust, Unilever plc and the University of Manchester.^1^ All the research studies described were approved by the University of Manchester Research Ethics Committee. From 1999-2002, those volunteers still actively participating in the project were invited to consent to collection of blood samples to extract DNA for genetic analyses, funded by AgeUK. Participants were genotyped for 599,011 common SNPs using the Illumina 610-Quadv1 chip (Illumina, Inc., San Diego, CA, USA.^2^ Stringent QC procedures were applied to the genotype data and 549,692 SNPs were retained. Individuals were excluded based on unresolved gender discrepancy, relatedness, call rate (≤0.95) and evidence of non-Caucasian descent. SNPs were included if they met the following conditions: call rate ≥0.98, minor allele frequency ≥0.01 and Hardy-Weinberg equilibrium test with *P* ≥ 0.001. The genotyping was funded by British Biotechnology and Biological Sciences Research Council.

A second set of 757 participants was recruited from Newcastle, UK following the same research protocol as Manchester.^1^ Volunteers were recruited by advertisements in local community centers, newspapers, radio, and television. Recruitment began in Newcastle in 1983/1984 with advertisements in local newspapers, radio and television. The Newcastle study attracted 2052 volunteers, 513 men aged from 49 to 86 years (mean age: 65.2 y, sd = 11.8 y) and 1539 women aged from 46 to 92 years (mean age: 67.4 y, sd = 14.3 y). All Newcastle participants were healthy, lived independently, and were able to make their way unaided to the University of Newcastle to take two different batteries of cognitive tests (described above). These were Test Battery 1 (TB1) on which they were screened at recruitment and on a second visit 2 years later, and Test Battery 2 (TB2), which, after the second visit, was alternated, with TB1 throughout the study. The University of Newcastle Research Ethics Committee approved all the research methods. Newcastle volunteers still actively participating in the project were invited to consent to collection of blood samples to extract DNA for genetic analyses, funded by AgeUK. Participants were genotyped for 599,011 common SNPs using the Illumina 610-Quadv1 chip (Illumina, Inc., San Diego, CA, USA).^2^ Stringent QC procedures were applied to the genotype data and 549,692 SNPs were retained.^2^ The genotyping was funded by British Biotechnology and Biological Sciences Research Council. Additional Manchester and Newcastle details are published previously.^1^

**ADNI (Alzheimer's Disease Neuroimaging Initiative)**

Data used in the preparation of this article were obtained from the Alzheimer’s Disease Neuroimaging Initiative (ADNI) database (adni.loni.usc.edu). ADNI was launched in 2003 by the National Institute on Aging (NIA), the National Institute of Biomedical Imaging and Bioengineering (NIBIB), the Food and Drug Administration (FDA), private pharmaceutical companies and non-profit organizations, as a $60 million, 5-year public-private partnership. The primary goal of ADNI has been to test whether serial magnetic resonance imaging (MRI), positron emission tomography (PET), other biological markers, and clinical and neuropsychological assessment can be combined to measure the progression of mild cognitive impairment (MCI) and early Alzheimer’s disease (AD). Determination of sensitive and specific markers of very early AD progression is intended to aid researchers and clinicians to develop new treatments and monitor their effectiveness, as well as lessen the time and cost of clinical trials. The Principal Investigator of this initiative is Michael W. Weiner, MD, VA Medical Center and University of California – San Francisco. ADNI is the result of efforts of many co-investigators from a broad range of academic institutions and private corporations, and subjects have been recruited from over 50 sites across the U.S. and Canada. The initial goal of ADNI was to recruit 800 subjects but ADNI has been followed by ADNI-GO and ADNI-2. To date these three protocols have recruited over 1500 adults, ages 55 to 90, to participate in the research, consisting of cognitively normal older individuals, people with early or late MCI, and people with early AD. The follow up duration of each group is specified in the protocols for ADNI-1, ADNI-2 and ADNI-GO. Subjects originally recruited for ADNI-1 and ADNI-GO had the option to be followed in ADNI-2. For up-to-date information, see www.adni-info.org.^3–5^

The current study included clinically stable healthy controls from ADNI1 and ADNI2GO. All ADNI participants provided written informed consent, and the institutional review board of each ADNI site approved study protocols. The neuropsychological tests selected for ADNI were mostly measures used by Alzheimer Disease Centers as part of a collective Uniform Data Set (UDS). The UDS is one part of a national collaborative research facilitated by the National Alzheimer's Coordinating Center and among approximately 30 Alzheimer's Disease Centers funded throughout the U.S. by NIA. ADNI1 was genotyped on the Illumina Human610-Quad BeadChip (620,901 SNP and CNV markers), and ADNI2GO was genotyped on the Illumina HumanOmniExpress BeadChip (730,525 SNP and CNV markers).

**ASPIS (Athens Study of Psychosis Proneness and Incidence of Schizophrenia)**

A detailed description of the ASPIS study has been reported previously.^6–8^ Briefly, ASPIS examined randomly selected young male conscripts aged 18 to 24 years from the Greek Air Force in their first two weeks of admission to the National Air Force Basic Training Center (Tripolis, Greece). All conscripts had received a standardized screening interview by a team of military doctors of different specialties in order to exclude serious medical conditions, including documented diagnosis of psychotic disorders and substance dependence, and individuals with such conditions were not admitted for military training. In all, 2029 eligible individuals provided a mouthwash sample for DNA extraction and completed a battery of computerized tasks measuring different aspects of neuropsychological and oculomotor performance. No conscript was excluded owing to medical conditions. All conscripts had already received a standardized screening interview by a team of army medical doctors of different specialties, and major medical conditions had been excluded. Con- scripts underwent an extensive interview of computerized neurocognitive abilities and a self-rated psychometric evaluation. After obtaining written informed consent, DNA was extracted from mouthwash samples. Written informed consent was obtained from every individual before participating to the study. The study protocol was approved by the University Mental Health Research Institute (Athens, Greece) and the Johns Hopkins University Institutional Review Boards.

**CAMH (Center for Addiction and Mental Health)**

CAMH participants were recruited under a study protocol was approved by the Research Ethics Board of CAMH, and all participants provided informed, written consent. All participants were identified as Caucasian based on self-reported ethnicity of three out of four grandparents. They were administered the Structured Clinical Interview for DSM-IV Disorders,^9^ and were interviewed by a psychiatrist to ensure diagnostic accuracy. Individuals with previous head trauma with loss of consciousness, neurological disorders, current or past substance dependence, and a history of a primary psychotic disorder in first-degree relatives were excluded. Eighty CAMH healthy control subjects completed cognitive testing and the genetic protocol.^10,11^ All participants were screened with the Mini Mental Status Exam for dementia (Folstein et al, 1975) and a urine toxicology screen. The Hand Dominance Questionnaire was used to examine handedness. All subjects underwent a battery of cognitive test that have been described previously^10^ that was administered over approximately 1.5 hours. This battery assessed a wide range of cognitive domains: executive function, working memory, immediate memory, delayed or episodic memory, attention, set-shifting, response inhibition, mental flexibility, visuospatial construction, processing speed, fine visuomotor, and motor skills.^10^

**CHS (Cardiovascular Health Study)**

CHS is a longitudinal observational study of risk factors for development and progression of heart disease and stroke sponsored by NIH/NHLBI. CHS was conducted across four U.S. field centers: Wake Forest University, Forsyth County, NC; University of California, Davis, Sacramento County, CA; Johns Hopkins University, Washington County, MD; and the University of Pittsburgh, Pittsburgh, PA. Eligible participants were sampled from Medicare eligibility lists in each area. Those eligible included all persons living in the household of each individual sampled from the Health Care Financing Administration (HCFA) sampling frame, who were 65 years or older at the time of examination, were noninstitutionalized, were expected to remain in the area for the next three years, and were able to give informed consent and did not require a proxy respondent at baseline. Approximately 5,888 study participants were recruited in CHS and have undergone extensive clinic examinations for evaluation of markers of subclinical cardiovascular disease.^12^ The original CHS cohort totaled 5,201 participants, and a second predominately African-American cohort (N = 687) was recruited in 1992. CHS participants were examined yearly from 1989 through 1999. Examination components included medical history questionnaires, measurement of ankle-brachial index, abdominal and carotid ultrasound studies, echocardiograms, ambulatory electrocardiograms, cerebral magnetic resonance imaging, spirometry, as well as brief cognitive testing.^13^ CHS collected blood samples for DNA extraction. Subsets of CHS participants have been genotyped in large-scale genotyping projects, including thousands of SNP genotypes for candidate gene regions as part of the NHLBI Candidate gene Association Resource (CARe) and genome-wide genotyping as part of the NHLBI SNP Typing for Association with Multiple Phenotypes from Existing Epidemiologic Data (STAMPEED). In the current study, we included Caucasian subjects of European ancestry based on whole-genome genotype data (N=2,931) with requisite SNP and cognitive data. Certain neuropsychological tests were added and/or discontinued over the course of the 11-year CHS evaluation period. As such, we divided CHS into three sub-studies (CHS1, based on the Year 11 evaluation; CHS2, based on the Year 6 evaluation; and CHS3, based on data collected at the intake/baseline evaluation).

**CNP (UCLA Consortium for Neuropsychiatric Phenomics)**

The UCLA Consortium for Neuropsychiatric Phenomics (CNP) is a large study funded by the NIH Roadmap Initiative that aims to facilitate discovery of the genetic and environmental bases of variation in psychological and neural system phenotypes, to elucidate the mechanisms that link the human genome to complex psychological syndromes, and to foster breakthroughs in the development of novel treatments for neuropsychiatric disorders. CNP comprises 8 components led by a team of 52 investigators representing diverse disciplines at the University of California, Los Angeles (UCLA), with five interlocking research projects supported by two research infrastructure cores and a coordinating center. Three research projects focus on clinical and laboratory approaches to understanding brain mechanisms underlying memory, response inhibition, and other behavioral functions disrupted in Schizophrenia, Bipolar Disorder, and Attention-Deficit/Hyperactivity Disorder. These projects examine variations in genetics, brain structure, brain function, and behavior in 2000 healthy people and 300 suffering from one of the target neuropsychiatric syndromes, and conduct parallel basic science experiments to unravel the biological mechanisms underlying these phenotypes. The participants, ages 21-50, were recruited by community advertisements from the Los Angeles area and completed extensive neuropsychological testing, in addition to fMRI scanning. All participants gave written informed consent according to the procedures approved by the Institutional Review Boards at UCLA and the Los Angeles County Department of Mental Health. To be included individuals had to be either "White, Not of Hispanic or Latino Origin" or "Hispanic or Latino, of Any Race" following NIH designations of racial and ethnic minority groups. However, for purposes of the present study, only subjects clustering with European ancestry individuals based on genetic data were include. For participants who spoke both English and Spanish, language for testing was determined by a verbal fluency test. Participants were screened for neurological disease, history of head injury with loss of consciousness or cognitive sequelae, use of psychoactive medications, substance dependence within past 6 months, history of major mental illness or ADHD, and current mood or anxiety disorder. Self-reported history of psychopathology was verified with the SCID-IV.^9^ Urinalysis was used to screen for drugs of abuse (cannabis, amphetamine, opioids, cocaine, benzodiazepines) on the day of testing and excluded if results were positive. CNP subjects were genotyped on the Illumina OmniExpress-12v1_A chip.

**DCC (Duke Cognition Cohort)**

Participants were recruited to be members of the DCC to study the genetics of normal variation in cognitive performance.^14,15^ Informed consent was obtained for all subjects as approved by the Duke University School of Medicine Institutional Review Board. All participants were healthy adult volunteers. Subjects were excluded if they had a MOCA score below 26, were taking a drug or combination of drugs that was decided by a pharmacist as likely to impact their cognition or be indicative of a cognitive impairment, were diagnosed with a serious neurological disorder, had a head injury resulting in memory problems, were diagnosed as learning disabled, or had a serious psychiatric history. Individuals with a blood relative already in the study were also excluded. Each subject donated 20 ml of blood or 5 ml saliva for DNA extraction. DNA was extracted using the QIAGEN (Venlo, The Netherlands) Autopure LS. The DNA was genotyped using Illumina (San Diego, CA, USA) HumanHap 300, Human610, HumanHap550, Human1M, or HumanCore genotyping chips at Duke University. Because of differences in genotyping platform, as well as differences in neuropsychological testing (described below), DCC is comprised of four sub-studies of only EUR participants (DCC1, n=498; DCC2, n=314; DCC3, n=234; and DCC4, n=147).

**DNS (Duke Neurogenetics Study)**

Participants were recruited as part of the Duke Neurogenetics Study (DNS), an ongoing study investigating biological mechanisms of individual differences in brain function and behavior.^16, 17^ Informed consent was obtained for all subjects as approved by the Duke University School of Medicine Institutional Review Board. All participants were healthy, young adult volunteers free of the following exclusion criteria included: (1) medical diagnoses of cancer, stroke, head injury with loss of consciousness, untreated migraine headaches, diabetes requiring insulin treatment, chronic kidney or liver disease, or lifetime history of psychotic symptoms; (2) use of psychotropic, glucocorticoid, or hypolipidemic medication; and (3) conditions affecting cerebral blood flow and metabolism (e.g. hypertension). The DNS seeks to establish broad variability in multiple behavioral phenotypes related to psychopathology, so participants were not excluded based on diagnosis of any past or current DSM-IV Axis I or Axis II disorder. No subjects were taking psychotropic medication at the time or at least 10 days prior to study participation. DNA was iolated from saliva derived from Oragene DNA self-collection kits (DNA Genotek) customized for 23andMe (www.23andme.com). DNA extraction and genotyping were performed through 23andMe by the National Genomics Institute (NGI), a CLIA-certified clinical laboratory and subsidiary of Laboratory Corporation of America. One of two different Illumina arrays with custom content was used to provide genome-wide SNP data: the HumanOmniExpress or HumanOmniExpress-24.^18-21^

**DUBLIN (****Galway and Dublin, Ireland)**

Participants were right-handed, Irish (i.e. Irish paternal and maternal grandparents) adults who were recruited from the general population at two sites in Ireland (Galway and Dublin) though outpatient clinics (for patients) and local media advertising (for healthy participants).^22^ Participants were screened for MRI safety criteria, as well as scientific and ethical criteria for inclusion in neuroimaging. Healthy participant sampling at the Dublin site included individuals involved in the Trinity College Biobank project [as described elsewhere;^23^]. Participants provided written, informed consent in accordance with local ethics committee guidelines. The sample consisted of 150 healthy individuals (mean age (years)=33.49; 84 male), of which ~80% were evaluated at.^22,23^ Genetics analysis was carried out using DNA obtained from blood samples or saliva samples that were collected using Oragene DNA self-collection kits (DNA Genotek). The Dublin study was genotyped on two different arrays, the Affymetrix 6.0 array (DUBLIN1) and the Illumina Exome array (DUBLIN2). DUBLIN1 and DUBLIN2 were evaluated using slightly different neuropsychological batteries (described below).

**FHS (Framingham Heart Study)**

FHS is a community-based, prospective study initiated in 1948 to identify risk factors for cardiovascular disease (CVD).^24^ FHS has followed 3 generations of participants comprised of (1) the Original Cohort, (2) the Offspring Cohort, followed since 1971 and consisting of the biological children of the Original Cohort and Offspring spouses, and (3) the Third Generation Cohort, which includes children from members of the Offspring Cohort, who have been followed since 2000.^24–26^ DNA has been collected from blood samples and from immortalized cell lines obtained from the FHS Original Cohort participants, members of the Offspring Cohort and the Third Generation Cohort. In the current study, we included a total of 5,360 FHS participants across the three cohorts divided into two groups (FHS1 and FHS2) based on the available neurocognitive data. FHS1 and FHS2 were genotyped on the Affymetrix GeneChip Human Mapping 500K Array Set, which is comprised of two arrays, the ~262,000 marker Nsp array and the ~238,000 marker Sty array. The research protocols of the Framingham Heart Study are reviewed annually by the Institutional Review Board of the Boston University Medical Center and by the Observational Studies Monitoring Board of the National Heart, Lung and Blood Institute. Since 1971, written consent has been obtained from participants before each examination, and all subjects participating in the DNA study have provided written, informed consent.

**GCAP (NIMH Genes, Cognition and Psychosis Program)**

GCAP participants were 639 healthy volunteers (GCAP1) and 325 unaffected siblings (GCAP2) from the NIMH Clinical Brain Disorder Branch (CBDB) Sibling Study of Schizophrenia Genetics.^27^ GCAP participants were between the ages of 18 and 61 years and were Caucasians of self-identified European descent (genotype data was used to either confirm or refute self-reported European ancestry). Rule-out of a psychiatric diagnosis was made independently by two psychiatrists/psychologists using the Structured Clinical Interview for DSM-IV Axis I disorders (SCID-I) and Structured Clinical Interview for DSM-IV Axis II disorders (SCID-II).^28^ Healthy volunteers in the GCAP study were excluded if they had first-degree relatives with schizophrenia spectrum disorders, if they were currently diagnosed with an Axis I disorder, or if they were taking neuroleptic medication. Unaffected siblings were excluded if they had a history of a psychotic spectrum disorder or schizotypal/schizoid personality disorder. Participants were also excluded if he/she had a history of serious head trauma, alcohol or drug abuse within the previous 6 months, IQ less than 70, or evidence of learning disability. All participants provided written informed consent. GCAP participants were genotyped on Illumina HumanHap550K, HumanHap610-Quad, or HumanOmni2.5S microarrays at the NIMH Clinical Brain Disorders Branch.^27^ After quality control procedures, 278,675 SNPs were available for subsequent genome-wide imputation for the GCAP cohort.

**GENADA (Genotype-Phenotype Associations in Alzheimer's Disease)**

GENADA is a multi-site study funded by GlaxoSmithKline, Inc. to study the genetics of Alzheimer's disease (AD). As previously described,^4,29,30^ this Canadian dataset comprised almost 1,000 patients with AD and almost 1,000 non-demented control subjects recruited from nine memory referral clinics in Canada between June 4, 2002 and March 30, 2005. All study participants voluntarily provided an informed and signed consent by self and/or legal representative. To date, data from 801 cases with probable AD and 782 controls without a family history of dementia were available for download from dbGaP; however, only Caucasian healthy controls with adequate SNP and cognitive data were included in the current COGENT analysis (N=768). Subjects were genotyped on the Affymetrix GeneChip Human Mapping 500K Array Set. The Mapping 500K platform is comprised of 2 arrays, the ~262,000 marker Nsp array and the ~238,000 marker Sty array. Combined, the two arrays yielded 500,568 SNPs.

**HBCS (Helsinki Birth Cohort Study)**

HBCS is composed of 8,760 individuals born between the years 1934-44 in one of the two main maternity hospitals in Helsinki, Finland. Between 2001 and 2003, a randomly selected sample of 928 males and 1075 females participated in a clinical follow-up study with a focus on cardiovascular, metabolic and reproductive health, cognitive function and depressive symptoms. Of those with genotype data, 320 men participated in assessment of cognitive functions in 2009 at the mean age of 67.7 years (SD: 2.3), which was the sample included in COGENT. The research plan of the HBCS was approved by the Institutional Review Board of the National Public Health Institute and all participants have signed an informed consent. HBCS samples were genotyped on the Illumina Infinium 610K Quad chip by the Wellcome Trust Sanger Institute, Cambridge, UK using standard procedures.

**IBG (Institute for Behavioral Genetics)**

The IBG samples were collected as part of two population based twin registries: the Colorado Longitudinal Twin Sample (LTS) and the Colorado Twin Sample (CTS)^31^. Inclusion criteria were that individuals be twins who lived in the area; there were no exclusion criteria with respect to IQ or psychiatric illness. Written informed consent (or assent from minors with consent from parent/guardian) was obtained from all participants. IBG youths of European ancestry were included (N=299). IBG samples were genotyped on the Affymetrix 6.0 SNP chip.

**LBC1936 (Lothian Birth Cohort 1936 Study)**

LBC1936 consists of 1,091 relatively healthy individuals first assessed on cognitive and medical traits at about 70 years of age. They were born in 1936, most took part in the Scottish Mental Survey of 1947, and almost all lived independently in the Lothian region of Scotland. A full description of participant recruitment and testing can be found elsewhere.^32^ Ethical approval for all projects was obtained from Scotland’s Multicentre Research Ethics Committee and the Lothian Research Ethics Committee. LBC1936 used venesected whole blood for DNA extraction. Participants were genotyped for 599,011 common SNPs using the Illumina 610-Quadv1 chip.^2^ Stringent QC procedures were applied to the genotype data and 549,692 SNPs were retained. Individuals were excluded from LBC1936 based on unresolved gender discrepancy, relatedness, call rate (≤0.95) and evidence of non-Caucasian descent. SNPs were included in the LBC1936 data if they met the following conditions: call rate ≥0.98, minor allele frequency ≥0.01 and Hardy-Weinberg equilibrium test with *P* ≥ 0.001.

**LLFS (****Long Life Family Study)**

LLFS is an international family-based cohort study designed to examine genetic, behavioral and environmental factors associated with exceptional survival traits.^33^ LLFS enrolled 4,559 long-lived probands and their siblings (n=1,445), their offspring (n=2,329) and spouse controls (n=785).^33^ The recruitment of families into the LLFS focused on selecting families with multiple exceptionally old living individuals. Families were recruited through elderly probands (generally in their 90’s) who self-reported on the survival history of their parents and siblings, and based on this information, families which showed clustering of exceptional survival were recruited. LLFS probands resided in the catchment areas of four Field Centers (Boston University, Columbia University, University of Pittsburgh, and University of Southern Denmark). Recruited family members were phenotyped through extensive in-home visits by teams of technicians who traveled all over the USA and Denmark. LLFS blood assays were centrally processed at a Laboratory Core at the University of Minnesota, and study protocols were standardized, monitored and coordinated through a Data Management Coordinating Center at Washington University St. Louis. A total of 4,953 LLFS participants were phenotyped in all major domains of healthy aging including cognition. Of these, 4,815 gave dbGaP sharing permission and had sufficient DNA for GWAS genotyping. LLFS subjects were genotyped on the Illumina 2.5M HumanOmni array, and genotypes were called using Bead Studio. To assess Mendelian errors on autosomal chromosomes, LOKIv3 was run on family data and removed 3,647 SNPs with enough Mendelian errors to be considered outlier SNPs. For SNPs that had Mendel errors, but not enough to be considered outlier SNPs, calls for that SNP were set to missing within each family that had a Mendel error which occurred 153,363 times in the data. Autosomal marker data was removed for 18 individuals who had an autosomal SNP call rate <97.5%, which were considered outliers as compared to the rest of the population. As a final familial QC check, Graphical Representation of Relationships (GRR) was used to check familial relationships based on Identity-by-State; corrections to the family relationships were made as warranted by the data. Quality control procedures for SNPs included eliminating SNPs with a call rate less than 98% (n=83,774 however 1,188 of these were Mendelian outlier SNPs). Applying both the call rate and Mendelian error criteria, 86,233 autosomal SNPs were removed, leaving ≈ 2 million SNPs passing these QC criteria. Additional SNP QC included the following: MAF <1%; deviation from Hardy-Weinberg equilibrium at *p* < 1E-06; if there was an allelic mismatch with 1000HG, and if the SNP was not present in 1000 HG. After these QC procedures, genotypes remained for 4,667 European ancestry participants. In the current study, we only included Caucasian subjects of European ancestry, which resulted in a sample of 4,081 individuals for whom SNP and cognitive data were available.

**LOAD (****Late Onset Alzheimer's Disease Family Study)**

The NIA-sponsored LOAD Family AD Study is an extensive effort to ascertain well-characterized families and patients with and without AD.^34^ The goal of LOAD was to identify and recruit families with two or more siblings with the late-onset form of AD and a cohort of unrelated, non-demented controls similar in age and ethnic background, and to make the clinical and genetic data available to qualified investigators. A set of 1,074 Caucasian control individuals of European ancestry with genetic and neuropsychological data from LOAD were included in the current COGENT analysis. As previously described,^34^ the recruitment criteria included a family with multiple members affected with late-onset AD that could provide clinical information and a biological sample for DNA extraction. The proband had to have a diagnosis of definite or probable late-onset AD with onset after 60 years of age, and a full sibling with definite, probable, or possible late-onset AD with onset after 60 years of age. A third biologically-related family member was required (first-, second-, or third-degree relative) of the affected sibling pairs and 60 years or older if unaffected, or 50 years or older if diagnosed as having late-onset AD or mild cognitive impairment. Unaffected persons were required to have had documented cognitive testing and clinical examination results to verify the clinical designation. LOAD participants were genotyped using the Illumina assay protocol with hybridization to Illumina Infinium II Human 610Quadv1_B Beadchips, conducted by the Center for Inherited Disease Research (CIDR).^34,35^

**LOGOS (Learning on Genetics of Schizophrenia Spectrum)**

The LOGOS project recruited 1540 randomly selected young male conscripts from the Greek Army (mean age 22.13; range 18–44) between June 2008 and July 2011 at the Military Training Camp of Candidate, Supply Army officers (SEAP) in Heraklion, Crete. Following public presentation of the study’s methods and goals in each consecutive series of new conscripts, all participants willing to volunteer received a detailed information sheet and gave written informed consent before screening. All subjects were thoroughly screened for past or current physical and mental health status by the army medical authorities, the study nurse and a trained research psychologist. They underwent a Mini-International Neuropsychiatric Interview,^36^ and were tested on a single occasion at some point during their 2 months military training in this establishment. Inclusion criteria were recent (last two months) conscript status in the camp and written informed consent. Exclusion criteria were left-handedness (n=150), personal history of head trauma, medical and neurological conditions (n=68), personal history of DSM-IV Axis I disorders (n=95), current use of prescribed drugs or a positive recreational drug screen (n=0) and a hearing test (n=53). On the basis of these criteria, and after 47 subjects who dropped out, cognitive and genetic data were available for 866 subjects. The LOGOS study was approved by the Ethics Committee of the University of Crete, the Executive Army Bureau, and the Bureau for the Protection of Personal and Sensitive Data of the Greek State. LOGOS was genotyped on Illumina HumanOmniExpress array.

**MCTFR (Minnesota Center for Twin and Family Research)**

Data used in the current study was accessed as part of the MCTFR Genome-Wide Association Study of Behavioral Disinhibition. This is an epidemiological study of substance abuse and related psychopathology in which the subjects were drawn from the Minnesota Center for Twin and Family Research. The MCTFR is a 20-year, longitudinal, community-representative study conducted at the University of Minnesota and approved by the University of Minnesota Institutional Review Board continuously since inception. It is in part a longitudinal study of two cohorts of adolescent twins and their parents. It additionally includes a parallel longitudinal study of adolescent adoptive siblings, biologically related siblings, and their parents. Over 1,500 twin families and 350 adoptive and biological sibling families have been studied, with follow-up assessments occurring approximately every 3 years. The MCTFR gathered detailed, standardized data on study participants including DSM-IIIR and DSM-IV diagnostic interview and questionnaire data. For the Genome-Wide Association Study of Behavioral Disinhibition, parental intake data plus adolescent data gathered closest to the proband child’s 17th birthday (between ages 16.5 and 21) was used.^37^ The MCTFR twin-family sample was ascertained through Minnesota birth records. The adoptive-family sample was ascertained from infant placements made by the three largest private adoption agencies in Minnesota. The non-adoptive controls were ascertained through Minnesota state birth records and selected to have a pair of siblings of comparable age and gender to the adoptive sibling pairs. Eligibility requirements for the adoptive families included having, at the time of the intake assessment, an adopted adolescent between the ages of 11 and 21 who had been placed permanently in the adoptive home prior to the age of 2 years and a second adolescent in the home who was not biologically related to the adopted adolescent and who was no more than 5 years different in age. The second child could be biologically related to one or both of the parents or could, like the first child, have been adopted and placed prior to the age of 2 years. Additional eligibility requirements, which applied to all subjects, included living within a day drive of the University of Minnesota laboratory and not having any physical or mental disability that would preclude completing the day-long, in-person intake assessment. To be included in this analysis, the subject must additionally have been willing to make a blood or saliva donation.

**MUNICH (Munich, Germany)**

Two independent samples from Munich, Germany were contributed by Dr. Dan Rujescu and colleagues. The first group of participants (MUNICH1) were randomly selected from the general population of Munich, Germany, and contacted by mail. To exclude subjects with central neurological diseases and psychotic disorders or subjects who had first-degree relatives with psychotic disorders, several screenings were conducted before the volunteers were enrolled in the study. First, subjects who responded were initially screened by telephone for the absence of neuropsychiatric disorders. Second, detailed medical and psychiatric histories were assessed for both themselves and their first-degree relatives by using a semi-structured interview. Third, if no exclusion criteria were fulfilled, subjects were invited to a comprehensive interview including the Structured Clinical Interview for DSM-IV Axis I Disorders–Patient Edition^38^ and the Structured Clinical Interview for DSM-IV Axis II Personality Disorders^39^ to validate the absence of any lifetime psychotic disorder. Additionally, the Family History Assessment Module^40^ was conducted to exclude psychotic disorders among first-degree relatives. A neurological examination was also conducted to exclude subjects with current central nervous system impairment. In volunteers older than 60 years, the Mini-Mental Status Test^41^ was performed to exclude subjects with possible cognitive impairment. The first Munich sample was genotyped on the Illumina OmniExpress chip.

A second set of Munich participants (MUNICH2) were also available for analysis. MUNICH2 consisted of 538 adults aged 19-72 who were demographically similar to the first sample. The second sample was recruited by the Psychiatric Clinic and Polyclinic of Ludwig Maximilians University, and included subjects of Caucasian descent negative for severe somatic and psychiatric disorders as well as suicidal behavior, history of head injury or neurological diseases. Absence of somatic disorders was evaluated by a semi-structured interview, absence of mental disorders and suicidal behavior with the Structured Clinical Interview for DSM-IV.^42^u Additional details of the Munich samples are published.^43^ The second Munich cohort was genotyped on either the Illumina HumanHap300 array or the HumanHap550-Quad array.^44^ All Munich participants provided written informed consent, and DNA was extracted from whole blood samples.

**NCNG (****Norwegian Cognitive NeuroGenetics Cohort)**

NCNG study participants were recruited through newspaper advertisements in the Oslo and Bergen urban areas of Norway. All participants were interviewed and probed for past or present neurological or psychiatric diseases known to affect the central nervous system, and for history of substance abuse. Any person with a history of treatment for any of these conditions was excluded from the sample. Participants should have completed basic education with no history of learning deficits; persons who, after initial inclusion, on subsequent testing scored more than one standard deviation below their age norm on intelligence or memory were excluded. Furthermore, persons with a score on a depression inventory indicating a previously undiagnosed depressive illness were excluded. The participants were native speakers of Norwegian. The project plan was approved by the regional ethical committee for medical research. Permission to obtain and store blood samples for genotyping in a biobank and to establish a registry with relevant information was granted by the Norwegian Department of Health. All participants gave their informed consent for participation, which included donation of a blood sample, DNA extraction and genotyping, and storage of the remaining blood sample in a biobank. NCNG genotyping has previously been described in detail.^2^ NCNG DNA samples were newly extracted from blood using the Qiagen Gentra Autopure LS system (Qiagen, Valencia, CA, USA). They were genotyped on the Illumina Human610-Quad Beadchip, and 554,225 SNPs were retained following QC.^2^

**PNC (****Philadelphia Neurodevelopmental Cohort)**

Participants were from the Philadelphia Neurodevelopmental Cohort (PNC) of the University of Pennsylvania Neurodevelopmental Genomics Study and the Children’s Hospital of Philadelphia (CHOP).^45–47^ NIH/NIMH funded PNC through the American Reinvestment and Recovery Act of 2009 (ARRA), and it is a collaborative research project between the Brain Behavior Laboratory at the University of Pennsylvania and the Center for Applied Genomics at the Children’s Hospital of Philadelphia. PNC consists of youths aged 8-21 years who volunteered to participate in genomic studies of complex pediatric disorders.^45–47^ As per published PNC reports, participants were first mailed a letter that described the study, followed by a scripted telephone call to establish that the individual was still interested in participation and was able to meet the minimal inclusion criteria. Inclusion criteria included (a) able to provide signed informed consent (for participants under age 18 assent and parental consent were required); (b) English proficiency; and (c) physically and cognitively able to participate in an interview and computerized neurocognitive testing. The overall sample consisted of children who came for pediatric care, gave blood for genomic studies, and consented to be contacted for future studies. Cognitive and psychiatric assessments were conducted at home (68.8% of participants) or in the laboratory (31.2%) depending on family preference. All participants underwent clinical assessment, including a neuropsychiatric structured interview and review of electronic medical records.^45–47^ PNC participants completed the Penn Computerized Neurocognitive Battery (Penn CNB) to assess cognition.^46,47^ Valid Penn CNB data from 8,526 PNC participants was downloaded via dbGaP. Samples were genotyped at the Center for Applied Genomics (CAG) at Children's Hospital of Philadelphia. In total, six SNP arrays were used to genotype the full cohort of 8,741 youths. The PNC subjects of European ancestry were genotyped on four different Illumina SNP chips, and using genome-wide genotype data, we determined that 4,711 of these subjects were of European ancestry with valid CNB data. Support for the collection of the data sets was provided by grant RC2MH089983 awarded to Raquel Gur and RC2MH089924 awarded to Hakon Hakonarson. All subjects were recruited through the Center for Applied Genomics at The Children’s Hospital in Philadelphia.

**TOP (Thematic Organized Psychosis Research Study)**

Participants were recruited in two waves as part of a large ongoing study on schizophrenia and bipolar disorder, the Thematic Organized Psychosis Research (TOP) study, which is run from the University Hospitals in Oslo, Norway. Two sub-studies (TOP1 and TOP2) were included in COGENT, and all subjects self-reported Norwegian ancestry, and PCA of an allele-sharing distance matrix across all subjects did not suggest any non-European ancestry genetic outliers.^48^. The healthy participants were randomly selected from national statistical records from the same catchment area and contacted by letter inviting them to participate. The healthy sample was screened with interview and with the Primary Care Evaluation of Mental Disorders (PRIME-MD)^49^, and subjects were excluded if they or any close relatives had a history of a severe psychiatric disorder (schizophrenia, bipolar disorder and major depression), or substance abuse or dependency in the last three months. Exclusion criteria for all groups were: IQ score below 70, hospitalized head injury, neurological disorder, unstable or uncontrolled medical condition that interferes with brain function (including hypothyroidism, uncontrolled hypertension and diabetes), and/or outside the age range 17-65 years. To assure valid neurocognitive test performance, all participants had to have Norwegian as their first language or have received their compulsory schooling in Norway, and had to score ≥15 on the forced recognition trial in the California Verbal Learning Test (CVLT-II)^50^. All participants gave written informed consent, and the study was approved by the Regional Committee for Medical Research Ethics and the Norwegian Data Inspectorate, and the Biobank was approved by the Health Department. In TOP1, DNA was collected and participants were genotyped on the Affymetrix 6.0 array and 597,198 SNPs passed quality control filters as previously reported.^51,52^ In TOP2, DNA was collected and participants were genotyped on the Illumina OmniExpress array and 605K SNPs passed quality control filters.

**ZHH (****Zucker Hillside Hospital)**

Participants from the New York metropolitan area were recruited through advertisements, word of mouth, referrals, and study registries. Participants had no history of a current DSM-IV Axis I major mood or psychotic disorder as assessed by structured diagnostic interview.^9^ Other exclusion criteria included: (1) intellectual or learning disability; and (2) significant medical illness that could affect brain structure and/or function. Written informed consent was obtained from all participants prior to neurocognitive testing. This study was approved by the Institutional Review Board of the North Shore – Long Island Jewish Health System. ZHH participants provided blood samples for DNA extraction. DNA samples were genotyped on approximately 1M SNPs using the Illumina Omni-1Quad platform. All quality-control procedures were performed in SVS version 7.3.1 (GoldenHelix Inc), except for cryptic identity and cryptic relatedness, which were performed in Plink. Following QC, 803,582 high-quality autosomal SNPs were available for analysis.^53^

**B. Neurocognitive Domains and Tests**

Scores on the following cognitive ability tests were used to create the general cognitive function component using principal components analysis. Subtests covered the core neuropsychological domains of interest including crystallized knowledge, fluid reasoning, attention, executive function, working memory, episodic memory, processing speed, language, visuospatial and a few other domain of interest.

Abbreviations: WMS (Wechsler Memory Scales, any version); WAIS (Wechsler Adult Intelligence Scales, any version); WASI (Wechsler Abbreviated Scale of Intelligence); TMT (Trail Making Test, A & B unless otherwise specified); CANTAB (The Cambridge Neuropsychological Test Automated Battery)

**ACPRC**

ACPRC Manchester and Newcastle participants were cognitively evaluated twice about five years apart. Evaluations took place across two 90-minute sessions using two alternating neuropsychological test batteries between the periods of 1983 to 2003. Test Battery 1 (TB1), Session 1, covered the domains of Fluid Reasoning (Alice Heim AH4 Intelligence Tests, Parts 1 and 2) and Crystallized Knowledge (Mill Hill Vocabulary Test, Part A [synonyms] and Part B [definitions]). TB1, Session 2, covered Verbal Learning and Memory (Verbal Free Recall of 30 Words Test and Cumulative Verbal Recall of 15 Words Test) and Nonverbal Learning and Memory (Pictorial Recognition Memory Test). Test Battery 2 (TB2), Session 1, covered the domains of Fluid Reasoning (Cattell Culture Fair Test), Crystallized Knowledge (WAIS Vocabulary Test), and Processing Speed (Savage Alphabet Coding Test). TB2, Session 2, covered Processing Speed (Visual Search for Letters Test), Verbal Learning and Memory (Verbal Free Recall for 10 Words Test and Propositions about People Test), and Nonverbal Learning and Memory (Memory for Shapes and Location Test and the Memory Circle Test).^1,2^

**ADNI**

Measures used in ADNI included American National Adult Reading Test (ANART; total errors [reverse scored]); WMS Logical Memory (encoding plus delayed recall total score); Rey Auditory Verbal Learning Test (RAVLT; encoding plus delayed recall total score); Animal Fluency (total words in 60 seconds); Trail Making Test B (TMTB; number of seconds to complete test); Clock Drawing (total score); and Boston Naming Test (BNT; total correct).^3-5^

**ASPIS**

As described previously, ^3-5^ measures included: Raven Progressive Matrices Test (Raven Matrices; raw score); Continuous Performance Task, Identical Pairs version (CPT-IP; d-prime score); Verbal N-Back working memory task (Verbal NBack; total accuracy); Spatial N-Back working memory task (Spatial NBack; total accuracy).^6^ Psychometric data were unavailable, as only composite (principal component) scores were provided.

**CAMH**

Available measures included: Wechsler Test for Adult Reading (WTAR) (Holdnack, 2001); RBANS Word list memory, Story memory, Figure memory, Letter-number span, Digit span, Trails B, Letter cancellation, Digit symbol coding, Letter fluency, Animal fluency, Line orientation, Stroop color-word.

**CHS**

Beginning in 1988-1989, all CHS participants completed the Modified Mini-Mental State Examination (3MSE; total score up to 100)^54^ and the Digit Symbol Substitution Test (DSST; symbols correctly coded in 90 seconds) from the WAIS-R^55^ at their annual visits; the Benton Visual Retention Test (BVRT; number of designs of 10 correctly drawn after 10 second exposure with stimulus covered and immediate reproduction from memory tested)^56^ was added for those tested between 1994 and 1998.^57^ At Year 11 of the study, the Trail Making Test was added to the battery. We designated participants who completed the full Year 11 battery (3MSE, DSST, BVRT and TMT) as CHS1 (n=1569). A second set of participants completed the 3MSE, DSST and BVRT at Year 6, which was designated as CHS2 (n=779). A third subset of participants completed the 3MSE, DSST, Walk Test and Grip Strength at baseline, designated as CHS3 (n=583).

**CNP**

Measures included: WASI Vocabulary, WASI Matrix Reasoning, WMS Digit Span, WMS Letter-Number Span, WMS Spatial Span and WMS Visual Reproduction, CVLT, and Choice Reaction Time (RT) task.

**DCC**

DCC participants took either the CANTAB battery or a traditional cognitive testing battery or both, as described below. Both batteries were administered to all participants in a private room under supervision of a trained administrator who read the instructions from a script. The traditional battery took approximately 30 min and comprised the following tests: Trail Making Test^58^ assesses rapid simple sequencing (Trails A) and complex sequencing, requiring the participant to follow a sequential pattern while shifting cognitive sets (Trails B); Controlled Oral Word Association (COWA; Multilingual Aphasia Examination)^59^ measures lexical fluency across three letters; Animal Fluency is a brief measure of semantic fluency, animals named in 60 seconds; Processing Speed subtests of the Wechsler Adult Intelligence Scale-III (WAIS-III)^60^ Digit-Symbol substitution, which assesses psychomotor sequencing, and Symbol Search, which measures scanning and target identification; Digit Span subtest of the WAIS-III^60^ measures attention and concentration as reflected by digit span forward and backward; Stroop Test^61^ measures sensitivity to interference through trials with changing task demands (reading, color identification, and response inhibition); and Green Prose Recall^62^ quantifies immediate and 30-minute delayed recall of contextually organized stories. The CANTAB battery took approximately 1 h and comprised the following tests: paired associates learning (PAL), spatial working memory (SWM), verbal recall (VRM) intra-extradimensional set shifting (IED), rapid visual processing (RVP), pattern recognition memory (PRM), spatial span (SSP) and spatial recognition memory (SRM). Further details of these tests can be found at the CANTAB web site (http://www.cantab.com/science/cantab-tests-all.asp). To reduce practice time and ceiling effects, modified versions of PAL, SWM, PRM, SSP, SRM, VRM were used for some subjects, as described in detail in Need *et al*.^14^

**DNS**

As described previously,^27^ DNS participants were administered a broad neuropsychological battery comprised of the following domains and tests: Processing Speed (Trail Making Test A & B); Attention & Working Memory (Digit Span Forward, Digit Span Backward, Digit Span Reordering, and the Paced Auditory Serial Addition Test [PASAT]); Phonemic Fluency (Letters [FAS]); Semantic Fluency (Categories [animals]); Crystallized Knowledge (WASI Vocabulary); and Fluid Reasoning (WASI Matrix Reasoning).

**DUBLIN**

Dublin participants were administered the WTAR, as well as vocabulary, block design, similarities and matrix reasoning from the WASI.

**FHS**

Neuropsychological domains and tests administered in main FHS1 sample were the following: Premorbid Ability (WRAT-3 Word Reading); Verbal Reasoning (WAIS Similarities); Verbal Learning & Memory (Logical Memory and Verbal Paired Associates from the WMS); Nonverbal Learning & Memory (Visual Reproduction from the WMS); Visuoperceptual Integration (Hooper Visual Orientation Test); Processing Speed (Trails A & B); Attention & Working Memory (Digit Span Forward and Digit Span Backward); and Phonemic Fluency (Letters [FAS]). For more information on FHS cognition data, see reference^26^. For the second FHS2 subcohort, six cognitive measures were available for analysis including three scores from the CERAD memory test (total, recall and retention)^63^ and three scores from the Victoria Stroop test,^64^ of which dot time, color time and interference were included in the analysis.

**GCAP**

Cognitive variables selected for GCAP (a) represented key domains of performance impairment in schizophrenia, (b) documented impairment in probands and unaffected siblings of probands, and (c) showed good distributional characteristics.^65^ Domains and tests available were the following: Premorbid Ability (WRAT-3 Word Reading); Crystallized Knowledge (WAIS-R Similarities); Fluid Reasoning (WAIS-R Picture Completion); Working Memory (WAIS-R Arithmetic and Digit Span Backward); Processing Speed (WAIS-R Digit Symbol Coding and Trails A & B); Episodic Memory (Logical Memory Immediate and Delayed Recall); Verbal Fluency (Phonemic [FAS] and Categorical [animals, fruits and vegetables]); Visuoperceptual Integration (Benton Judgment of Line Orientation); and Set-shifting (Wisconsin Card Sorting Task). GCAP1 also had adequate data from Letter-Number Sequencing and the N-back, and GCAP2 had adequate data on Verbal Paired Associates and Visual Reproduction.

**GENADA**

The neuropsychological battery used in GENADA consisted of the Mattis Dementia Rating Scale, 1^st^ Edition (DRS);^66–68^ the Mini-Mental Status Exam (MMSE);^69^ and the Clock Drawing Test.^70^ The DRS was designed to test core areas of cognition most affected in AD including Attention, Conceptualization, Construction, Initiation & Perseveration, and Memory. The MMSE is similarly comprised of core neurocognitive domains including Attention, Construction, Language, Memory, Orientation, and Praxis. Clock Drawing is a classic test of Visuospatial Integration and Construction.

**HBCS**

The cognitive functions test scores were obtained from the Finnish Defense Forces Basic Ability Test, developed by the Finnish Defense Forces Education Development Center. The test battery and its psychometric properties are described in detail elsewhere.^71^ In brief, the ability test battery, which was designed to measure general ability and logical thinking, is composed of verbal, arithmetic, and visuospatial reasoning subtests. Each subtest is timed and consists of 40 multiple-choice questions that are ordered by difficulty. Correct answers were summed to obtain a test score. The verbal and arithmetic subtests comprise four types of questions. In the verbal reasoning test, the subject has to choose synonyms or antonyms of a given word, select a word belonging to the same category as a given word pair, identify which word of a word list does not belong in the group, and discern similar relations between word pairs. In the arithmetic reasoning test, the subject has to complete a series of numbers that have been arranged to follow a certain rule, to solve verbally expressed short problems, to complete simple arithmetic operations, and to choose similar relations between pairs of numbers. The visuospatial reasoning subtest comprises a set of matrices containing a pattern problem with one part removed; it is analogous to Raven’s Progressive Matrices. The subject is asked to decide which of the given single figures completes the matrix, and the test requires the subject to conceptualize spatial relations ranging from the very obvious to the very abstract. Detailed information on the selection of the HBCS participants and on the study design can be found elsewhere.^72–74^

**IBG**

All IBG subjects were administered a test covering Crystallized Knowledge (Vocabulary) and Fluid Reasoning (Block Design) from the Wechsler Adult Intelligence Scale (WAIS-III)^60^ if aged 16 or older, or from the Wechsler Intelligence Scale for Children (WISC-III)^75^ if younger than 16 years of age. The two-subtest combination of Vocabulary plus Block Design has excellent reliability, correlates highly with the Full Scale IQ score over a wide age range, and is a good measure of *g*.^76^

**LBC1936**

The LBC1936 cognitive test battery was composed from measures from the Wechsler Adult Intelligence Scale-III, U.K. version (WAIS-III,UK).^77^ The WAIS-III subtests used in COGENT covered the following neuropsychological domains: Processing Speed (Digit Symbol Coding and Symbol Search); Fluid Reasoning (Block Design and Matrix Reasoning); and Working Memory (Digit Span Backwards and Letter-Number Sequencing).

**LLFS**

The LLFS neuropsychological battery covered the following domains and tests: Verbal Learning & Memory (Logical Memory Immediate and Delayed Recall); Attention and Working Memory (Digit Span Forward and Digit Span Backward); and Semantic Fluency (Categories [animals and vegetables]). Additional details of initial neuropsychological findings from LLFS have been reported recently.^78–80^

**LOAD**

As described previously,^34^ the LOAD cognitive test battery included the following domains and tests: Verbal Learning & Memory (Logical Memory Immediate and Delayed Recall); Attention and Working Memory (Digit Span Forward and Digit Span Backward); and Semantic Fluency (Categories [animals and vegetables]).

**LOGOS**

As described in detail previously^81^, subjects were administered three subtests of the Cambridge Neuropsychological Test Automated Battery^82^ namely, Spatial Working Memory (SWM), Stockings of Cambridge (SOC), a planning and problem solving test, and Rapid Visual Information Processing (RVIP), a sustained attention test similar to a CPT. These are nonverbal tests which were administered with the aid of a high-resolution touch-sensitive screen (Advantech) and/or a response key to all subjects in the same order. Visual working memory was assessed with the N-Back Sequential Letter Task^83^. Cognitive flexibility and problem solving were assessed using a computerized version of the Wisconsin Card Sorting Test (WCST)^84^. The Stroop Interference Test^61^ was used to measure the selection of appropriate response and interference. Subjects were administered the Iowa Gambling Task (IGT)^85^ to assess planning based on emotional processing and integration of incentive information for decision-making. Finally, we used the Word Lists subtest of the Wechsler Memory Scale^86^ to assess verbal learning and memory.

**MCTFR**

Neurocognitive data from the MCTFR study was available as a composite full-scale IQ (FSIQ) score. Measurement of FSIQ was included in the design of the intake assessment for most participants, by way of an abbreviated form of the Wechsler Intelligence Scale for Children-Revised (WISC-R) or Wechsler Adult Intelligence Scale-Revised (WAIS-R), as age-appropriate (that is, 16 or younger, and older than 16, respectively). The short forms consisted of two Performance subtests (Block Design and Picture Arrangement) and two Verbal subtests (Information and Vocabulary), the scaled scores on which were prorated to determine FSIQ. FSIQ estimates from this short form have been shown to correlate 0.94 with FSIQ from the complete test.^76^

**MUNICH**

MUNICH1 participants completed the full German version of the Wechsler Adult Intelligence Scale–Revised (WAIS-R) IQ battery, which is called the HAWIE-R in Germany.^87^ The German WAIS-R included four Crystallized Knowledge subtests (Information, Vocabulary, Similarities and Comprehension), four Fluid Reasoning subtests (Block Design, Object Assembly, Picture Completion and Picture Arrangement), two Working Memory subtests (Digit Span and Arithmetic) and one Processing Speed subtest (Digit Symbol Coding). MUNICH2 participants completed the same 11 subtests from the HAWIE-R/WAIS-R.^43,87^

**NCNG**

NCNG participants completed a battery of psychometric tests, assessing general cognition, memory, attention and speed of processing faculties. The recruitment procedure resulted in a cognitively normal sample, skewed towards the higher functioning intelligence range. General cognitive ability was generated using a hierarchy of principal components analysis (PCA) steps as previously described.^88^ The unrotated first component for three subtests from the California Verbal Learning Test-II^50^ defined a Memory factor. The first component from the four conditions of D-KEFS^89^ Color Word Interference Test defined a Speed factor. These two factor scores, together with the raw score from the Matrix Reasoning subscale of the Norwegian WASI,^90^ and the overall mean of median reaction times from a multiple choice reaction time task, were used as input for a further PCA, of which the unrotated first component defined the general cognitive ability factor.

**PNC**

PNC participants completed the Penn Computerized Neurocognitive Battery (Penn CNB).^45,46^ The Penn CNB assesses the following: Abstraction & Concept Formation (Conditional Exclusion Test); Verbal Reasoning (Verbal Reasoning Test); Nonverbal Reasoning (Matrix Reasoning Test); Attention (Continuous Performance Test); Working Memory (Letter N-back Test); Verbal Memory (Word Memory Test); Nonverbal Memory (Face Memory Test and Visual Object Learning Test); and Sensory-Motor Processing Speed (Motor Praxis Test and Finger Tapping Test). The Wide Range Assessment Test 4 (WRAT-4) was also administered and included in the current analysis. For all Penn CNB tests, total correct/accuracy was the primary dependent measure.

**TOP**

Neurocognitive assessment in TOP was carried out by psychologists trained in standardized neuropsychological testing. For TOP1, a 3-hour test battery (including measures of estimated premorbid IQ and adequate test effort) was administered in a fixed order with two breaks with refreshments. Attention & Working Memory were tested with the Digit Span Backward, Digit Span Forward and Letter Number components of the Wechsler Adult Intelligence Scale Third Edition (WAIS-III)^60^ and the simple RT and d-prime components of the Bergen *N*-back test.^91^ Verbal Fluency was tested with the Letter Fluency, Category Fluency and Category Switching components of the Delis-Kaplan Executive Function System (D-KEFS) Word Fluency Test, and Condition 1: Color Naming (CW-1), Condition 2: Word Reading components (CW-2), Condition 3: Inhibition (CW-3) and Condition 4: Inhibition/Switching components (CW-4) of the DKEFS Color-Word Interference Test.^85^ Psychomotor Speed was tested with the Digit Symbol Coding component of the WAIS-III^60^ and with the left and right hand average for the Grooved Pegboard Test.^92^ Learning and Memory (Verbal and Nonverbal) were tested with the California Verbal Learning Test Second Edition (CVLT-II),^50^ the Logical Memory I Recall Total Score of the Wechsler Memory Scale Third Edition (WMS-III),^93^ and the Long Term Memory component of the Rey Complex Figure Test (RCFT-LTM).^94^ General intelligence was tested with the Block Design, Matrix Reasoning, Similarities and Vocabulary components of the Wechsler Abbreviated Scale of Intelligence (WASI),^90^ and the National Adult Reading Test (NART).^95^ The

4-subtest estimated IQ from the WASI was strongly correlated (r=.67, p<10^-46^) with the

computed *g* utilized in the present study. A subset of these measures were administered to TOP2 participants including the NART, Vocabulary, Similarities, Block Design, Matrix Reasoning, Digit Span, Letter-Number Span, and Logical Memory.

**ZHH**

ZHH participants were recruited to serve as healthy comparison subjects for studies of patients with schizophrenia and other psychiatric disorders, and cognitive testing was mostly performed using the MATRICS Consensus Cognitive Battery (MCCB).^96,97^ The MCCB evaluates seven domains of cognitive function including: 1) Speed of Processing (BACS Digit Symbol Coding, Trail Making Test Part A and Semantic Fluency (Animals); 2) Attention/Vigilance (Continuous Performance Test - Identical Pairs version (CPT-IP); 3) Working Memory (Spatial Span and Letter-Number Span); 4) Verbal Learning (Hopkins Verbal Learning Test - Revised [HVLT-R]); 5) Visual Learning (Brief Visuospatial Memory Test - Revised [BVMT-R]); and 6) Reasoning & Problem Solving (NAB Mazes). The seventh MCCB domain, Social Cognition, was excluded from the current analysis, and three additional measures were added: Trails B, Phonemic Fluency (FAS) and WRAT-3 Word Reading.

**C. Phenotyping Procedures**

**I. Neuropsychological data quality control**

Phenotyping procedures followed protocols similar to what has already been published for COGENT.^98,99^ In general, only the most relevant variable from a given neuropsychological test was included. The majority of the time, this was the variable reflecting overall accuracy or total number of correct responses. For some tests (e.g., CPT tasks), we utilized signal detection theory^100^ to calculate d-prime rather than pure accuracy whenever accuracy was not normally distributed. Lastly, some of the reaction/response time data (e.g., Trail Making Test) had to be logarithmically transformed prior to analysis to better approximate a Gaussian distribution.

The cognitive phenotypes used in all 35 COGENT sub-studies were subjected to a series of quality control (QC) steps to ensure high quality general cognitive function “*g*” factor data was generated. (1) Raw cognitive variables were plotted several ways using SPSS version 21 (IBM SPSS Inc.) and the R statistical package (Version 3.1.1, “Sock it to Me”, release date 7/10/2014) for analysis. Histograms, normal probability plots, detrended normal probability plots and boxplots were examined for blatant outliers (e.g., incorrect values from data entry errors) and removed when necessary. To the extent possible, we used truly “raw” variables as opposed to already-transformed or normed variables for each dataset (e.g., the raw score instead of a T-score that was generated from a standardization sample). (2) Five Sex and Age variables were created and regressed out of every individual cognitive variable in each study: [i] Sex; [ii] Age; [iii] Age^2^ (the product of the age-centered variable); [iv] Sex × Age; and [v] Sex × Age^2^. (3) Dummy coding was used to create binary indicator variables for samples that had potential “batch effects” that needed to be controlled. For batch effects, the largest subcohort or batch within each study was used as the reference group. (4) The five age/sex variables (and batch variables if necessary) were regressed out of each individual cognitive test variable using linear regression, and the standardized residuals (transformed to *z*-scores [*M* = 0, *SD* = 1]) saved for subsequent analysis. (5) Each residualized cognitive variable was re-plotted as described above and “extreme outliers” removed. An extreme outlier was defined as a value whose distance from the nearest quartile was greater than 1.5 times the interquartile range (IQR) in either direction. In most cases, the extreme outliers that were flagged were z-scores of ±4.0. Lastly, cognitive tests missing for more than 10% of a given sample were excluded; individual participants within each study were then excluded if they were missing more than 20% of the data from his/her own neurocognitive battery.

**II. Calculation of the general cognitive function *g* factor**

Cronbach’s Alpha (α) coefficient was calculated using the R *psych* package^101^ to estimate reliability of the neurocognitive measures used in each sample, and by natural extension, the degree of factor saturation of the underlying latent general cognitive function trait that was captured using these measures.^102^ Tests that substantially reduced α were also dropped. As shown in Table S1, the average coefficient α was 70% across all 35 COGENT sub-studies, meaning *g* was reliably measured, and most of the variance in *g* was attributable to a general latent construct common to items in each test battery.^102^ The primary method for estimating each person’s *g* performance was principal components analysis (PCA). PCA is widely used and a valid method to capture general neurocognitive performance. PCA-based construction of the *g* factor followed the logic of prior COGENT studies.^98,99^ At least three neuropsychological tests (or at least two IQ-test subscales) were used to generate the first unrotated principal component factor score for each individual within each sample. On average, the first PCA accounted for 41% of the variance in cognitive performance.

**D. Genotyping Procedures**

**I. GWAS quality control**

All COGENT samples were genotyped on commercially available Affymetrix or Illumina SNP microarrays. We developed a semi-automated GWAS QC pipeline that was applied to all 35 sub-cohorts. QC parameters were set at the following cutoffs: MAF >1%; call rate ≥98%; sample call rate ≥98%; and Hardy–Weinberg equilibrium *P*>10^−6^.

*Population stratification.* GWAS data was pruned to generate a LD-independent variant set that was used to calculate population stratification parameters (e.g., inbreeding coefficients and identity-by-state [IBS] clustering), and to check sex assignment. Multidimensional scaling (MDS) axes were generated to identify ancestry outliers (i.e., non-European samples) and “nearest neighbor” estimates based on genetic clustering. The MDS axes were plotted versus ancestry data from the 1000 Genomes Project to identify individuals who did not cluster with the European ancestry clusters 1000G. Individuals were removed for excess autosomal heterozygosity (either reflecting DNA sample contamination or a sample that does not belong to the core population)^103^, sex mismatches if they could not be resolved, or for being a genetic outlier based on nearest neighbor analysis and MDS plotting.

*Cryptic Relatedness.* Next, we calculated genome-wide pairwise identity-by-descent (IBD) given identity-by-state (IBS) information within each sample. The proportion of alleles shared IBD0 and IBD1 were plotted to confirm known relatives and/or detect cryptic relatedness. Samples with IBD sharing greater than .125 were flagged for cryptic relatedness, except for subjects from known pedigrees in cohorts utilizing family-based ascertainment. To do so, one (randomly selected, or otherwise the individual with full neurocognitive data) of a pair of individuals who were unknowingly related in the sample of interest were removed. Several COGENT cohorts (e.g., FHS, LLFS and MCTFR) were known to comprise family members a priori; these studies were analyzed using MLMA (described below).

**II. Imputation**

After QC, all SNPs within a given cohort were strand-aligned to 1000G and imputed against the largest available reference panel, derived from 64,976 haplotypes characterized at 39,235,157 SNPs made available by the Haplotype Reference Consortium.^104^

**E. Statistical and Analytical Procedures**

**I. GWAS meta-analysis**

As described in the main text, the association between genome-wide allele dosage data and general cognitive function was completed separately within each COGENT sub-study. For cohorts in which subjects were unrelated, ordinary-least-squares (OLS) regression was used and analyses were performed in PLINK 1.9.^105^ The first 10 MDS factors were used as covariates to control for subtle population stratification effects in each sub-study. For samples in which subjects were known to comprise family members, we used mixed-linear-model association (MLMA) as implemented in BOLT-LMM.^106^ MLMA in BOLT-LMM accounts for relatedness structure through a correction specific to the structure of interest.^106^

GWAS results were generated within each study and then carried forward for meta-analysis using METAL.^107^ All analyses were conducted using two-tailed tests the METAL inverse-variance option.

**II. Gene analysis**

Individual SNP results from the meta-analysis were aggregated to conduct a gene analysis using MAGMA.^108^ SNPs were mapped to genes based on NCBI build 37.3 and defined by the start and stop site ±5 kb, resulting in 18,164 autosomal genes. A genome-wide significance threshold for gene-based associations was calculated using the Bonferroni method (α = .05/18,164; P=2.75×10−6).

**III. Genetic correlation analysis**

LD score regression analyses followed the methods described in Bulik-Sullivan *et al* using the LDSC software package.^109,110^ First, we estimated the intercept to assess if our comprehensive GWAS results exhibited signs of polygenicity vs population stratification. The LD Score regression method can disentangle inflation that is due to a true polygenic signal throughout the genome (which affects the slope of the LD Score regression) from inflation that is due to confounding biases such as cryptic relatedness and population stratification (which affects the intercept of the regression).^109–111^ LD Scores were computed with genotypes from the European-ancestry samples in the 1000 Genomes Project using only HapMap3 SNPs that impute well in European samples. Only HapMap3 SNPs with MAF > 0.01 were included in the LD score regression analyses. Running the LD score regression on these data, we estimated an intercept of 1.043 (S.E. = 0.0071), consistent with true polygenic signal throughout the genome.^111^

**F. Sources of Data for LD Score Regression**

For each quantitative trait or categorical diagnosis examined in the LDSC analyses, we list the source of the GWAS summary statistics below:

College attainment, years of education^111^ – SSGAC – http://ssgac.org/Data.php

Childhood cognitive performance^112, 113^ – CHIC – http://ssgac.org/Data.php

ADHD^114^ – PGC-ADD2 - www.med.unc.edu/pgc

Autism spectrum disorders^114^ – PGC-AUT - [www.med.unc.edu/pgc](http://www.med.unc.edu/pgc)

Alzheimer's disease^115^ – IGAP - <http://web.pasteur-lille.fr/en/recherche/u744/igap/igap_download.php>

Anorexia nervosa^114^ – PGC-AN - www.med.unc.edu/pgc

Bipolar disorder^114^ – PGC-BIP2 - www.med.unc.edu/pgc

Major depressive disorder^114^ – PGC-MDD2 - www.med.unc.edu/pgc

Schizophrenia^116^ – PGC-SCZ2 - www.med.unc.edu/pgc

Personality factors^117^ – GPC – http://www.tweelingenregister.org/GPC/

Smoking behaviors^118^ - TAG - <https://www.med.unc.edu/pgc/downloads>

Birth weight^119^ – EGG – http://egg-consortium.org/

Length at birth^120^ – EGG – http://egg-consortium.org/

Infant head circumference at birth^121^ – EGG – <http://egg-consortium.org/>

Subcortical brain volumes^122^ – ENIGMA – <http://enigma.loni.usc.edu/#genomics>

Intracranial and hippocampal volumes^123^ – ENIGMA – <http://enigma.loni.usc.edu/#genomics>

**2. Supplementary Figures**

Figure S1.

**
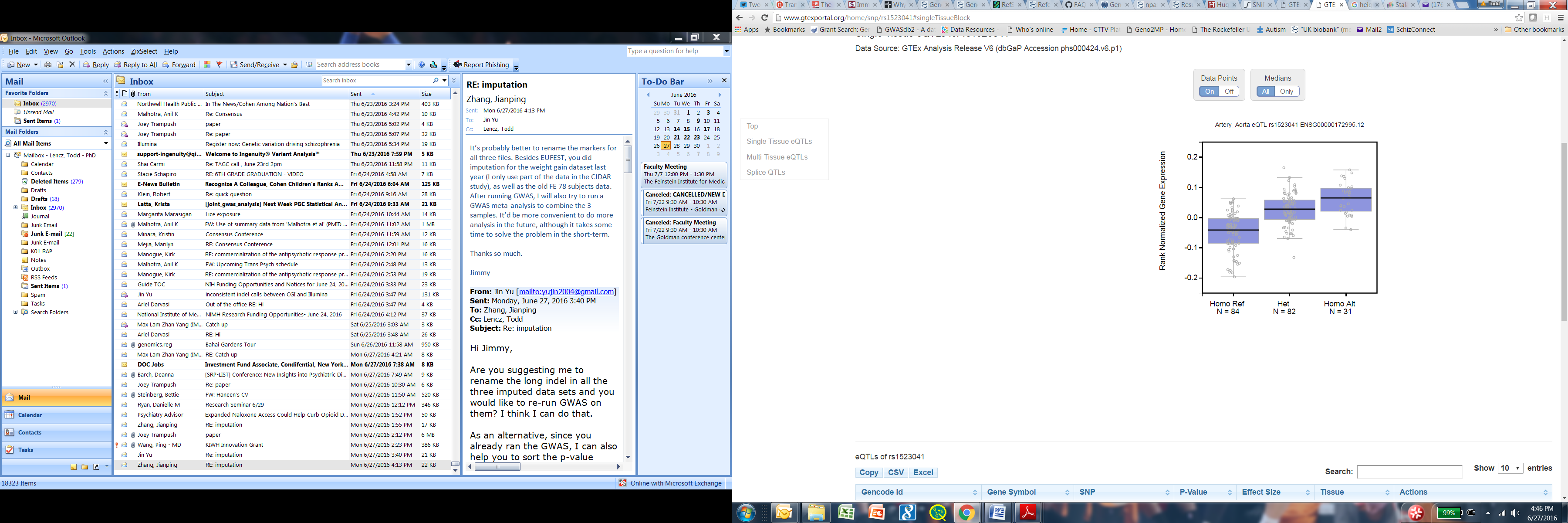
**

Figure S2.

**
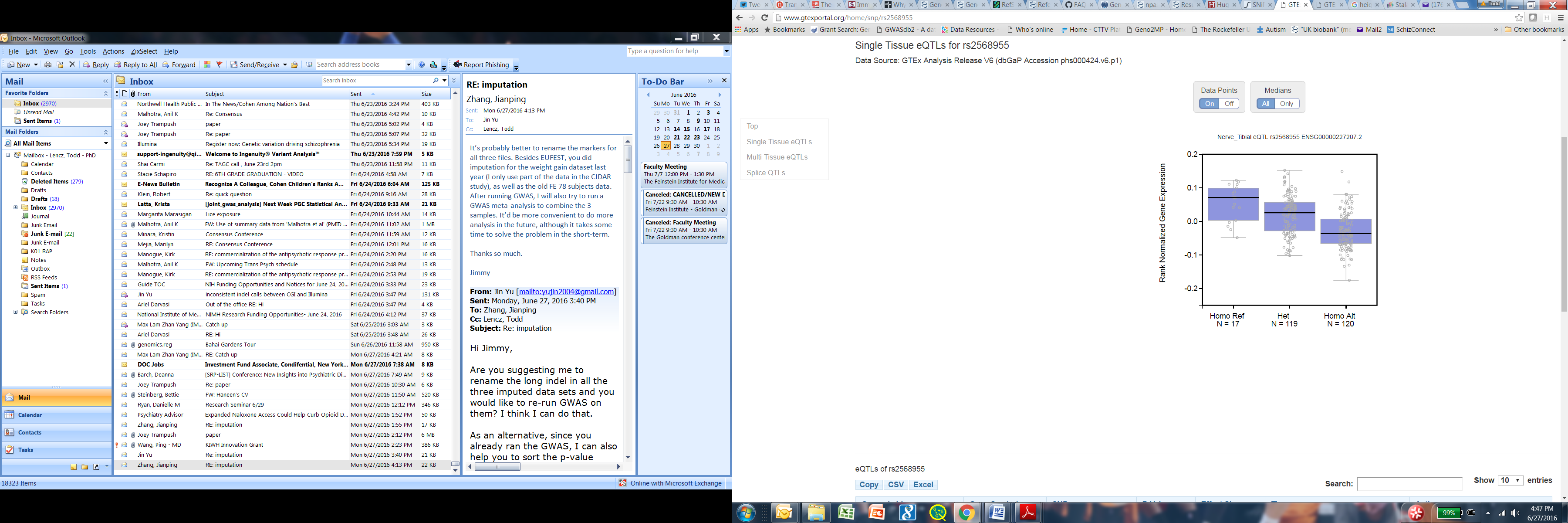
**

**3. Supplemental References**

1 Rabbitt PMA, McInnes L, Diggle P, Holland F, Bent N, Abson V *et al.* The University of Manchester Longitudinal Study of Cognition in Normal Healthy Old Age, 1983 through 2003. *Aging, Neuropsychol Cogn* 2004; **11**: 245–279.

2 Davies G, Tenesa A, Payton A, Yang J, Harris SE, Liewald D *et al.* Genome-wide association studies establish that human intelligence is highly heritable and polygenic. *Mol Psychiatry* 2011; **16**: 996–1005.

3 Mueller SG, Weiner MW, Thal LJ, Petersen RC, Jack CR, Jagust W *et al.* Ways toward an early diagnosis in Alzheimer’s disease: the Alzheimer's Disease Neuroimaging Initiative (ADNI). *Alzheimer’s Dement* 2005; **1**: 55–66.

4 Martinez-Mir A, González-Pérez A, Gayán J, Antúnez C, Marín J, Boada M *et al.* Genetic study of neurexin and neuroligin genes in Alzheimer’s disease. *J Alzheimer’s Dis* 2013; **35**: 403–12.

5 Morra JH, Tu Z, Apostolova LG, Green AE, Avedissian C, Madsen SK *et al.* Automated 3D mapping of hippocampal atrophy and its clinical correlates in 400 subjects with Alzheimer’s disease, mild cognitive impairment, and elderly controls. *Hum Brain Mapp* 2009; **30**: 2766–2788.

6 Smyrnis N, Avramopoulos D, Evdokimidis I, Stefanis CN, Tsekou H, Stefanis NC. Effect of schizotypy on cognitive performance and its tuning by COMT val158 met genotype variations in a large population of young men. *Biol Psychiatry* 2007; **61**: 845–53.

7 Hatzimanolis A, Bhatnagar P, Moes A, Wang R, Roussos P, Bitsios P, Stefanis CN, Pulver AE, Arking DE, Smyrnis N, Stefanis NC, Avramopoulos D. Common genetic variation and schizophrenia polygenic risk influence neurocognitive performance in young adulthood. *Am J Med Genet B Neuropsychiatr Genet.* 2015 Jul;168B(5):392-401.

8 Stefanis NC, Trikalinos TA, Avramopoulos D, Smyrnis N, Evdokimidis I, Ntzani EE *et al.* Impact of schizophrenia candidate genes on schizotypy and cognitive endophenotypes at the population level. *Biol Psychiatry* 2007; **62**: 784–92.

9 First MB, Spitzer RL, Gibbon M, Williams JB. *Structured Clinical Interview for DSM-IV Axis I Disorders–Non-Patient Edition.* New York Biometrics Research Dept, New York State Psychiatric Institute, 1995.

10 Voineskos AN, Rajji TK, Lobaugh NJ, Miranda D, Shenton ME, Kennedy JL *et al.* Age-related decline in white matter tract integrity and cognitive performance: a DTI tractography and structural equation modeling study. *Neurobiol Aging* 2012; **33**: 21–34.

11 Lett TA, Kennedy JL, Radhu N, Dominguez LG, Chakravarty MM, Nazeri A *et al.* Prefrontal white matter structure mediates the influence of GAD1 on working memory. *Neuropsychopharmacology* 2016; : 1–28.

12 Fried LP, Borhani NO, Enright P, Furberg CD, Gardin JM, Kronmal RA *et al.* The Cardiovascular Health Study: design and rationale. *Ann Epidemiol* 1991; **1**: 263–276.

13 Lopez OL, Kuller LH, Mehta PD, Becker JT, Gach HM, Sweet RA *et al.* Plasma amyloid levels and the risk of AD in normal subjects in the Cardiovascular Health Study. *Neurology* 2008; **70**: 1664–1671.

14 Need AC, Attix DK, McEvoy JM, Cirulli ET, Linney KL, Hunt P *et al.* A genome-wide study of common SNPs and CNVs in cognitive performance in the CANTAB. *Hum Mol Genet* 2009; **18**: 4650–61.

15 Cirulli ET, Kasperaviciūte D, Attix DK, Need AC, Ge D, Gibson G *et al.* Common genetic variation and performance on standardized cognitive tests. *Eur J Hum Genet* 2010; **18**: 815–20.

16 Nikolova YS, Koenen KC, Galea S, Wang C-M, Seney ML, Sibille E *et al.* Beyond genotype: serotonin transporter epigenetic modification predicts human brain function. *Nat Neurosci* 2014; **17**: 1153–1155

17 Scult MA, Trampush JW, Zhang F, Conley ED, Lencz T, Malhotra AK, *et al.* A common polymorphism in SCN2A predicts general cognitive ability through effects on PFC physiology. *J Cogn Neurosci.* 2015 Sep;27(9):1766-74.

18 Eriksson N, Macpherson JM, Tung JY, Hon LS, Naughton B, Saxonov S *et al.* Web-based, participant-driven studies yield novel genetic associations for common traits. *PLoS Genet* 2010; **6**: 1–20.

19 Tung JY, Do CB, Hinds DA, Kiefer AK, Macpherson JM, Chowdry AB *et al.* Efficient replication of over 180 genetic associations with self-reported medical data. *PLoS One* 2011; **6**. doi:10.1371/journal.pone.0023473.

20 Do CB, Tung JY, Dorfman E, Kiefer AK, Drabant EM, Francke U, *et al.* Web-based genomewide association study identifies two novel loci and a substantial genetic component for Parkinson's disease. *Plos Genetics,* 2011, 7: e1002141.

21 Hu Y, Shmygelska A, Tran D, Eriksson N, Tung J, Hinds D. GWAS of 89,283 individuals identifies genetic variants associated with being a morning person. *Nat Commun.* 2016, 7: 10448.

22 Rose EJ, Morris DW, Fahey C, Cannon D, McDonald C, Scanlon C *et al.* The mir-137 schizophrenia susceptibility variant rs1625579 does not predict variability in brain volume in a sample of schizophrenic patients and healthy individuals. *Am J Med Genet B Neuropsychiatr Genet* 2014; **165B**: 467–71.

23 Rose EJ, Donohoe G. Brain vs behavior: an effect size comparison of neuroimaging and cognitive studies of genetic risk for schizophrenia. *Schizophr Bull* 2013; **39**: 518–26.

24 Bangen KJ, Beiser A, Delano-Wood L, Nation DA, Lamar M, Libon DJ *et al.* APOE genotype modifies the relationship between midlife vascular risk factors and later cognitive decline. *J Stroke Cerebrovasc Dis* 2013; **22**: 1361–1369.

25 Feinleib M, Kannel WB, Garrison RJ, McNamara PM, Castelli WP. The Framingham offspring study. Design and preliminary data. *Prev Med (Baltim)* 1975; **4**: 518–525.

26 Seshadri S, Wolf PA, Beiser A, Elias MF, Au R, Kase CS *et al.* Stroke risk profile, brain volume, and cognitive function: the Framingham Offspring Study. *Neurology* 2004; **63**: 1591–1599.

27 Dickinson D, Straub RE, Trampush JW, Gao Y, Feng N, Xie B *et al.* Differential effects of common variants in SCN2A on general cognitive ability, brain physiology, and messenger RNA expression in schizophrenia cases and control individuals. *JAMA Psychiatry* 2014; **21205**. doi:10.1001/jamapsychiatry.2014.157.

28 First MB, Gibbon M. The Structured Clinical Interview for DSM-IV Axis I Disorders (SCID-I) and the Structured Clinical Interview for DSM-IV Axis II Disorders (SCID-II). In: *Comprehensive handbook of psychological assessment, Vol. 2: Personality assessment.* 2004, pp 134–143.

29 Li H, Wetten S, Li L, St Jean PL, Upmanyu R, Surh L *et al.* Candidate single-nucleotide polymorphisms from a genomewide association study of Alzheimer disease. *Arch Neurol* 2008; **65**: 45–53.

30 Hu X, Pickering E, Liu YC, Hall S, Fournier H, Katz E *et al.* Meta-analysis for genome-wide association study identifies multiple variants at the BIN1 locus associated with late-onset Alzheimer’s disease. *PLoS One* 2011; **6**: e16616.

31 Rhea S-A, Gross AA, Haberstick BC, Corley RP. Colorado Twin Registry. *Twin Res Hum Genet* 2006; **9**: 941–9.

32 Deary IJ, Gow AJ, Pattie A, Starr JM. Cohort profile: the Lothian Birth Cohorts of 1921 and 1936. *Int J Epidemiol* 2012; **41**: 1576–84.

33 Newman AB, Glynn NW, Taylor CA, Sebastiani P, Perls TT, Mayeux R *et al.* Health and function of participants in the Long Life Family Study: A comparison with other cohorts. *Aging (Albany NY)* 2011; **3**: 63–76.

34 Lee JH, Cheng R, Graff-Radford N, Foroud T, Mayeux R. Analyses of the National Institute on Aging Late-Onset Alzheimer’s Disease Family Study: implication of additional loci. *Arch Neurol* 2008; **65**: 1518–26.

35 Shi H, Belbin O, Medway C, Brown K, Kalsheker N, Carrasquillo M *et al.* Genetic variants influencing human aging from late-onset Alzheimer’s disease (LOAD) genome-wide association studies (GWAS). *Neurobiol Aging* 2012; **33**: 1849.e5–18.

36 Sheehan D V, Lecrubier Y, Sheehan KH, Amorim P, Janavs J, Weiller E *et al.* The Mini-International Neuropsychiatric Interview (M.I.N.I.): the development and validation of a structured diagnostic psychiatric interview for DSM-IV and ICD-10. *J Clin Psychiatry* 1998; **59 Suppl 2**: 22–33;quiz 34–57.

37 Kirkpatrick RM, McGue M, Iacono WG, Miller MB, Basu S. Results of a ‘GWAS Plus:’ General Cognitive Ability Is Substantially Heritable and Massively Polygenic. *PLoS One* 2014; **9**: e112390.

38 First MB, Spitzer RL, Gibbon M, Williams JB. Structured Clinical Interview for DSM-IV Axis I Disorders - Patient Edition. *New York Biometrics Res Dep New York State Psychiatr Inst* 1995.

39 First MB, Spitzer RL, Gibbon M, Williams BW. Structured Clinical Interview for DSM-IV Axis II Personality Disorders (SCID-II). *New York Biometrics Res Dep New York State Psychiatr Inst* 1990.

40 Rice JP, Reich T, Bucholz KK, Neuman RJ, Fishman R, Rochberg N *et al.* Comparison of direct interview and family history diagnoses of alcohol dependence. *Alcohol Clin Exp Res* 1995; **19**: 1018–23.

41 Kessler J, Folstein SE, Denzler P. Mini-Mental Status Test, German Version. *Weinheim, Ger Beltz* 1990.

42 Wittchen HU, Zaudig M, Fydrich T. *SCID. Structured Clinical Interview for DSM-IV Axis I and II. Manual.* Gottingen: Hogrefe, 1997.

43 Rujescu D, Hartmann a M, Gonnermann C, Möller H-J, Giegling I. M129V variation in the prion protein may influence cognitive performance. *Mol Psychiatry* 2003; **8**: 937–41.

44 Ising M, Mather K a, Zimmermann P, Brückl T, Höhne N, Heck A *et al.* Genetic effects on information processing speed are moderated by age - converging results from three samples. *Genes Brain Behav* 2014; : 501–507.

45 Gur RC, Calkins ME, Satterthwaite TD, Ruparel K, Bilker WB, Moore TM *et al.* Neurocognitive growth charting in psychosis spectrum youths. *JAMA psychiatry* 2014; **71**: 366–74.

46 Gur RE, Richard J, Calkins ME, Chiavacci R, Hansen JA, Bilker WB *et al.* Age group and sex differences in performance on a computerized neurocognitive battery in children age 8−21. Neuropsychology. 2012; **26**: 251–265.

47 Roalf DR, Gur RC, Ruparel K, Calkins ME, Satterthwaite TD, Bilker WB *et al.* Within-individual variability in neurocognitive performance: Age- and sex-related differences in children and youths from ages 8 to 21. *Neuropsychology* 2014; **28**: 506–18.

48 Bakken TE, Roddey JC, Djurovic S, Akshoomoff N, Amaral DG, Bloss CS *et al.* Association of common genetic variants in GPCPD1 with scaling of visual cortical surface area in humans. *Proc Natl Acad Sci U S A* 2012; **109**: 3985–90.

49 Spitzer RL, Williams JB, Kroenke K, Linzer M, DeGruy F V, Hahn SR *et al.* Utility of a new procedure for diagnosing mental disorders in primary care. The PRIME-MD 1000 study. *JAMA* 1994; **272**: 1749–56.

50 Delis DC, Kramer JH, Kaplan E, Ober BA. *California Verbal Learning Test (CVLT-II). Norwegian manual supplement.* Pearson Assessment: Stockholm, 2004.

51 Djurovic S, Gustafsson O, Mattingsdal M, Athanasiu L, Bjella T, Tesli M *et al.* A genome-wide association study of bipolar disorder in Norwegian individuals, followed by replication in Icelandic sample. *J Affect Disord* 2010; **126**: 312–316.

52 Athanasiu L, Mattingsdal M, Kähler AK, Brown A, Gustafsson O, Agartz I *et al.* Gene variants associated with schizophrenia in a Norwegian genome-wide study are replicated in a large European cohort. *J Psychiatr Res* 2010; **44**: 748–753.

53 Malhotra AK, Correll CU, Chowdhury NI, Müller DJ, Gregersen PK, Lee AT *et al.* Association between common variants near the melanocortin 4 receptor gene and severe antipsychotic drug-induced weight gain. *Arch Gen Psychiatry* 2012; **69**: 904–12.

54 Teng EL, Chui HC. The Modified Mini-Mental State (3MS) examination. *J Clin Psychiatry* 1987; **48**: 314–318.

55 Wechsler D. *Wechsler Adult Intelligence Scale - Revised.* Psychological Corp: New York, 1981.

56 Benton AL. The visual retention test as a constructional praxis task. *Confin Neurol* 1962; **22**: 141–155.

57 Lopez OL, Jagust WJ, DeKosky ST, Becker JT, Fitzpatrick A, Dulberg C *et al.* Prevalence and classification of mild cognitive impairment in the Cardiovascular Health Study Cognition Study: part 1. *Arch Neurol* 2003; **60**: 1385–1389.

58 USArmy. Army Individual Test Battery. In Manual of Directions and Scoring. 1944.

59 Benton A, Hamsher K, Sivan A. *Multilingual Aphasia Examination: Manual of Instructions.* AJA Associates Inc.: Iowa City, 1978.

60 Wechsler D. *Wechsler Adult Intelligence Scale-Third Edition (WAIS-III).* The Psychological Corporation: San Antonio, 1997.

61 Golden C. The measurement of creativity by the Stroop color and word test. *J Pers Assess* 1975; **39**: 502–6.

62 Green P. *Story Recall Test.* Green’s Publishing: Edmonton, 2005.

63 Lamberty GJ, Kennedy CM, Flashman LA. Clinical utility of the CERAD word list memory test. *Appl Neuropsychol* 1995; **2**: 170–3.

64 Troyer AK, Leach L, Strauss E. Aging and response inhibition: Normative data for the Victoria Stroop Test. *Neuropsychol Dev Cogn B Aging Neuropsychol Cogn* 2006; **13**: 20–35.

65 Dickinson D, Goldberg TE, Gold JM, Elvevåg B, Weinberger DR. Cognitive factor structure and invariance in people with schizophrenia, their unaffected siblings, and controls. *Schizophr Bull* 2011; **37**: 1157–67.

66 Smith GE, Ivnik RJ, Malec JF, Kokmen E, Tangalos E, Petersen RC. Psychometric Properties of the Mattis Dementia Rating Scale. Assessment. 1994; **1**: 123–131.

67 Schmidt R, Freidl W, Fazekas F, Reinhart B, Grieshofer P, Koch M *et al.* The Mattis Dementia Rating Scale: normative data from 1,001 healthy volunteers. *Neurology* 1994; **44**: 964–966.

68 Lucas JA, Ivnik RJ, Smith GE, Bohac DL, Tangalos EG, Kokmen E *et al.* Normative data for the Mattis Dementia Rating Scale. *J Clin Exp Neuropsychol* 1998; **20**: 536–47.

69 Folstein MF, Folstein SE, McHugh PR. ‘Mini-mental state’. A practical method for grading the cognitive state of patients for the clinician. *J Psychiatr Res* 1975; **12**: 189–198.

70 Battersby WS, Bender MB, Pollack M, Kahn RL. Unilateral spatial agnosia (inattention) in patients with cerebral lesions. *Brain* 1956; **79**: 68–93.

71 Tiihonen J, Haukka J, Henriksson M, Cannon M, Kieseppä T, Laaksonen I *et al.* Premorbid intellectual functioning in bipolar disorder and schizophrenia: results from a cohort study of male conscripts. *Am J Psychiatry* 2005; **162**: 1904–10.

72 Barker DJP, Osmond C, Forsén TJ, Kajantie E, Eriksson JG. Trajectories of growth among children who have coronary events as adults. *N Engl J Med* 2005; **353**: 1802–9.

73 Eriksson JG, Osmond C, Kajantie E, Forsén TJ, Barker DJP. Patterns of growth among children who later develop type 2 diabetes or its risk factors. *Diabetologia* 2006; **49**: 2853–8.

74 Rikkönen K, Pesonen A-K, Heinonen K, Lahti J, Kajantie E, Forsén T *et al.* Infant growth and hostility in adult life. *Psychosom Med* 2008; **70**: 306–13.

75 Wechsler D. *Wechsler Intelligence Scale for Children-Third Edition (WISC-III).* The Psychological Corporation: San Antonio, 1991.

76 Sattler JM. *Assessment of Children: Cognitive Applications*. 4th ed. Jerome M. Sattler: La Mesa, 2001.

77 Wechsler D. *WAIS-III UK Administration and Scoring Manual.* Psychological Corporation: London, 1998.

78 Barral S, Cosentino S, Costa R, Matteini A, Christensen K, Andersen SL *et al.* Cognitive function in families with exceptional survival. *Neurobiol Aging* 2012; **33**: 619.e1–7.

79 Barral S, Cosentino S, Costa R, Andersen SL, Christensen K, Eckfeldt JH *et al.* Exceptional memory performance in the Long Life Family Study. *Neurobiol Aging* 2013; **34**: 2445–8.

80 Cosentino S, Schupf N, Christensen K, Andersen SL, Newman A, Mayeux R. Reduced prevalence of cognitive impairment in families with exceptional longevity. *JAMA Neurol* 2013; **70**: 867–74.

81 Roussos P, Giakoumaki SG, Georgakopoulos A, Robakis NK, Bitsios P. The CACNA1C and ANK3 risk alleles impact on affective personality traits and startle reactivity but not on cognition or gating in healthy males. *Bipolar Disord* 2011; **13**: 250–9.

82 Sahakian BJ, Owen AM. Computerized assessment in neuropsychiatry using CANTAB: discussion paper. *J R Soc Med* 1992; **85**: 399–402.

83 Cohen JD, Perlstein WM, Braver TS, Nystrom LE, Noll DC, Jonides J *et al.* Temporal dynamics of brain activation during a working memory task. *Nature* 1997; **386**: 604–8.

84 Nelson HE. A modified card sorting test sensitive to frontal lobe defects. *Cortex* 1976; **12**: 313–24.

85 Bechara A, Damasio AR, Damasio H, Anderson SW. Insensitivity to future consequences following damage to human prefrontal cortex. *Cognition* 1994; **50**: 7–15.

86 Wechsler D. *WMS-III: Technical Manual.* The Psychological Corporation: San Antonio, 1997.

87 Tewes U. Hamburg-Wechsler Intelligenztest für Erwachsene (HAWIE-R). *Göttingen, Ger Hogrefe* 1991.

88 Espeseth, T., Christoforou, A., Lundervold, A. J., Steen, V. M., Le Hellard, S., & Reinvang, I. (2012). Imaging and cognitive genetics: The Norwegian Cognitive NeuroGenetics sample. *Twin Research and Human Genetics, 15,* 442-452.

89 Delis DC, Kaplan E, Kramer J. *The Delis-Kaplan Executive Function System (D-KEFS). Norwegian manual.* Pearson Assessment: Stockholm, 2005.

90 Wechsler D. *Wechsler Abbreviated Scale of Intelligence (WASI). Norwegian manual supplement.* Pearson Assessment: Stockholm, 2007.

91 Haatveit BC, Sundet K, Hugdahl K, Ueland T, Melle I, Andreassen OA. The validity of d prime as a working memory index: results from the ‘Bergen n-back task’. *J Clin Exp Neuropsychol* 2010; **32**: 871–80.

92 Klove H. Clinical neuropsychology. In: Forster FM (ed). *The Medical Clinics of North America*. Saunders: New York, 1963.

93 Wechsler D. *Wechsler Memory Scale-third edition (WMS-III). Norwegian manual.* Pearson Assessment: Stockholm, 2007.

94 Meyers JE, Meyers KR. *Rey Complex Figure Test and Recognition Trial: Professional manual.* Psychological Assessment Resources: Lutz, FL, 1995.

95 Vaskinn A, Sundet K. Estimating premorbid IQ: A Norwegian version of National Adult Reading Test. *J Nor Psychol Assoc* 2001; **38**: 1133–1140.

96 Kern RS, Nuechterlein KH, Green MF, Baade LE, Fenton WS, Gold JM *et al.* The MATRICS Consensus Cognitive Battery, part 2: co-norming and standardization. *Am J Psychiatry* 2008; **165**: 214–20.

97 Nuechterlein KH, Green MF, Kern RS, Baade LE, Barch DM, Cohen JD *et al.* The MATRICS consensus cognitive battery, part 1: Test selection, reliability, and validity. *Am J Psychiatry* 2008; **165**: 203–213.

98 Trampush JW, Lencz T, Knowles E, Davies G, Guha S, Pe’er I *et al.* Independent evidence for an association between general cognitive ability and a genetic locus for educational attainment. *Am J Med Genet Part B Neuropsychiatr Genet* 2015; : n/a–n/a.

99 Lencz T, Knowles E, Davies G, Guha S, Liewald DC, Starr JM *et al.* Molecular genetic evidence for overlap between general cognitive ability and risk for schizophrenia: a report from the Cognitive Genomics consorTium (COGENT). *Mol Psychiatry* 2013. doi:10.1038/mp.2013.166.

100 Stanislaw H, Todorov N. Calculation of signal detection theory measures. *Behav Res Methods Instrum Comput* 1999; **31**: 137–149.

101 Revelle W. psych: Procedures for Personality and Psychological Research. 2014.

102 Zinbarg RE, Revelle W, Yovel I, Li W. Cronbach’s α, Revelle’s β, and Mcdonald’s ωH: their relations with each other and two alternative conceptualizations of reliability. *Psychometrika* 2005; **70**: 123–133.

103 Weale ME. Quality control for genome-wide association studies. In: Barnes MR, Breen G (eds). *Genetic Variation: Methods and Protocols, Methods in Molecular Biology*. Humana Press, 2010, pp 341–372.

104 McCarthy S, Das S, Kretzschmar W, Delaneau O, Wood AR, Teumer A, *et al.* A reference panel of 64,976 haplotypes for genotype imputation. Nat Genet. 2016 Aug 22. [Epub ahead of print].

105 Chang CC, Chow CC, Tellier LC, Vattikuti S, Purcell SM, Lee JJ. Second-generation PLINK: rising to the challenge of larger and richer datasets. *Gigascience* 2015; **4**: 7.

106 Loh P, Tucker G, Bulik-Sullivan BK, Vilhjálmsson BJ, Finucane HK, Salem RM *et al.* Efficient Bayesian mixed-model analysis increases association power in large cohorts. *Nat Genet* 2015; **47**: 284–290.

107 Willer CJ, Li Y, Abecasis GR. METAL: Fast and efficient meta-analysis of genomewide association scans. *Bioinformatics* 2010; **26**: 2190–2191.

108 de Leeuw CA, Mooij JM, Heskes T, Posthuma D. MAGMA: generalized gene-set analysis of GWAS data. *PLoS Comput Biol* 2015; **11**: 1–19.

109 Bulik-Sullivan BK, Loh P-R, Finucane HK, Ripke S, Yang J, Consortium SWG of the PG *et al.* LD Score regression distinguishes confounding from polygenicity in genome-wide association studies. *Nat Genet* 2015; **advance on**: 291–295.

110 Bulik-Sullivan B, Finucane HK, Anttila V, Gusev A, Day FR, Loh P-R *et al.* An atlas of genetic correlations across human diseases and traits. *Nat Genet* 2015; **47**: 1236–1241.

111 Okbay A, Beauchamp JP, Fontana MA, Lee JJ, Pers TH, Rietveld CA *et al.* Genome-wide association study identifies 74 loci associated with educational attainment. *Nature* 2016.

112 Rietveld CA, Esko T, Davies G, Pers TH, Turley P, Benyamin B et al. Common genetic variants associated with cognitive performance identified using the proxy-phenotype method. Proc Natl Acad Sci U S A 2014.

113 Benyamin B, Pourcain B, Davis OS, Davies G, Hansell NK, Brion M-J a *et al.* Childhood intelligence is heritable, highly polygenic and associated with FNBP1L. *Mol Psychiatry* 2014; **19**: 253–8.

114 Cross-Disorder Group of the Psychiatric Genomics et al., Genetic relationship between five psychiatric disorders estimated from genome-wide SNPs. Nat Genet 45, 984 (Sep, 2013).

115 Jean-Charles Lambert et al. Meta-analysis of 74,046 individuals identifies 11 new susceptibility loci for Alzheimer's disease. Nature Genetics, 2013, 45 : 1452–1458.

116 Working Group of the Psychiatric Genomics Consortium. Biological insights from 108 schizophrenia-associated genetic loci. Nature. 2014 Jul 24;511(7510):421-7.

117 De Moor et al. Meta-analysis of genome-wide association studies for personality. Molecular Psychiatry, 2012; 17, 337-349

118 Tobacco and Genetics Consortium. Genome-wide meta-analyses identify multiple loci associated with smoking behavior. Nat Genet 42, 441 (May, 2010).

119 Horikoshi M, Beaumont RN, Day FR, Warrington NM, Kooijman MN, et al. Genome-wide associations for birth weight and correlations with adult disease. Nature. 2016 Sep 28;538(7624):248-252.

120 van der Valk RJ, Kreiner-Møller E, Kooijman MN, Guxens M, Stergiakouli E, Sääf A, et al. A novel common variant in DCST2 is associated with length in early life and height in adulthood. Hum Mol Genet. 2015 Feb 15;24(4):1155-68.

121 Taal HR, St Pourcain B, Thiering E, Das S, Mook-Kanamori DO, Warrington NM, Kaakinen M, et al. Common variants at 12q15 and 12q24 are associated with infant head circumference. Nat Genet. 2012 Apr 15;44(5):532-8.

122 Hibar DP, Stein JL, Renteria ME, Arias-Vasquez A, Desrivières S, Jahanshad N, et al. Common genetic variants influence human subcortical brain structures. Nature. 2015 Apr 9;520(7546):224-9

123 Stein JL, Medland SE, Vasquez AA, Hibar DP, Senstad RE, Winkler AM, et al. Identification of common variants associated with human hippocampal and intracranial volumes. Nat Genet. 2012 Apr 15;44(5):552-61.
