# Supplementary material for: GWAS meta-analysis reveals novel loci and genetic correlates for general cognitive function: a report from the COGENT consortium
Source: Mol Psychiatry. 2017 Jan 17;22(3):336–45. doi: 10.1038/mp.2016.244 (PMC5322272; doi:10.1038/mp.2016.244)
Supplement: Supplementary Figure Legends [file mp2016244x3.docx]

Supplementary Figure S1. Relationship between rs1523041 and expression of the *ARPP21* gene; data derived from the GTEx^43^ database. Normalized gene expression values are shown for each of three genotypes at rs1523041: homozygotes for the reference allele (CC genotype); heterozygotes (CG genotype); and homozygotes for the alternate allele (GG genotype). Note that we observed that the reference (C) allele at rs1523041 was strongly associated with better cognitive performance; this allele drives lower expression of the *ARPP21* gene.

Supplementary Figure S2. Relationship between rs2568955 and expression of *RPL31P12*; data derived from the GTEx^43^ database. Normalized gene expression values are shown for each of three genotypes at rs2568955: homozygotes for the reference allele (TT genotype—note that the reference allele is the minor allele for this SNP); heterozygotes (CT genotype); and homozygotes for the alternate allele (CC genotype). We found that the minor (T) allele of rs2568955 was associated with poorer cognitive performance, and this allele is associated with greater expression of *RPL31P12*.
